# The structural model of Zika virus RNA-dependent RNA polymerase in complex with RNA for rational design of novel nucleotide inhibitors

**DOI:** 10.1038/s41598-018-29459-7

**Published:** 2018-07-24

**Authors:** Jakub Šebera, Anna Dubankova, Vladimír Sychrovský, Daniel Ruzek, Evzen Boura, Radim Nencka

**Affiliations:** 1 Gilead Sciences Research Centre at IOCB Prague, Institute of Organic Chemistry and Biochemistry of the Czech Academy of Sciences, Praha, Czech Republic; 2 Institute of Organic Chemistry and Biochemistry of the Czech Academy of Sciences, Praha, Czech Republic; 3 Veterinary Research Institute, Hudcova 70, CZ-62100 Brno, Czech Republic; 4 Institute of Parasitology, Biology Centre of the Czech Academy of Sciences, Branisovska 31, CZ-37005 Ceske Budejovice, Czech Republic

## Abstract

Zika virus is a global health threat due to significantly elevated risk of fetus malformations in infected pregnant women. Currently, neither an effective therapy nor a prophylactic vaccination is available for clinical use, desperately necessitating novel therapeutics and approaches to obtain them. Here, we present a structural model of the Zika virus RNA-dependent RNA polymerase (ZIKV RdRp) in complex with template and nascent RNAs, Mg^2+^ ions and accessing nucleoside triphosphate. The model allowed for docking studies aimed at effective pre-screening of potential inhibitors of ZIKV RdRp. Applicability of the structural model for docking studies was illustrated with the NITD008 artificial nucleotide that is known to effectively inhibit the function of the ZIKV RdRp. The ZIKV RdRp – RNA structural model is provided for all possible variations of the nascent RNA bases pairs to enhance its general utility in docking and modelling experiments. The developed model makes the rational design of novel nucleosides and nucleotide analogues feasible and thus provides a solid platform for the development of advanced antiviral therapy.

## Introduction

Zika virus (ZIKV) is an emerging mosquito-borne member of the family *Flaviviridae*, genus *Flavivirus*. This genus includes several major human pathogens like the dengue virus, Japanese encephalitis virus, West Nile virus, and tick-borne encephalitis virus. Virtually the entire human population lives where at least one flavivirus species is endemic^1^. The ZIKV was first identified in 1947 in Uganda^2^. Until its sudden emergence in Brazil in 2015 and explosive spread through South America, Central America, and the Caribbean, the virus had been thought to produce a rare and mild, self-limiting disease in humans, with common symptoms that include fever, rash, joint pain, and conjunctivitis^3^. However, during the recent outbreak it was discovered that ZIKV infection is linked to adverse pregnancy and birth defects, most notably microcephaly and other serious brain anomalies^4^. In adults, ZIKV can cause rare but severe neurological complications, including Guillain-Barré syndrome (a poorly understood immunopathological disease affecting the nervous system that may lead to paralysis or death), acute disseminated encephalomyelitis (an immune-mediated inflammatory demyelinating condition that predominately affects the white matter of the brain and spinal cord), acute myelitis, meningitis or encephalitis^5–9^ with fatal consequences in isolated cases^7,10^. During the most recent outbreak in Brazil, an estimated 440 000–1 300 000 cases of ZIKV infection have been reported^11^.

Flaviviruses are small, enveloped viruses carrying genomes which consist of non-segmented single-stranded positive-sense RNA (+ RNA). The genome contains one large open reading frame (ORF), which is flanked with untranslated regions^12^. Viral RNA bears a type-1 cap structure (m7GpppAm) at the 5′-end and has no poly(A) tail at the 3′-end. The ORF encodes a single polyprotein that is subsequently processed into three structural (C, prM/M, and E) and seven non-structural (NS1, NS2A, NS2B, NS3, NS4A, NS4B, and NS5) proteins^12^. Central enzymatic activities related to virus replication are encoded by NS3 and NS5 proteins. NS3 is the second largest viral protein and possesses both the protease and helicase activities, with NS2B serving as a cofactor for the protease^13^. The NS5 protein is the largest virus-encoded protein, comprised of about 900 amino acids (AAs), and is also the most highly conserved protein among flaviviruses^14^. Approximately 270 AAs at the N-terminus form the methyltransferase (MTase) domain, and approximately 620 AAs at the C-terminus form the RNA-dependent RNA polymerase (RdRp)^15^. The MTase is involved in type 1 cap synthesis at the 5′-end of the viral RNA. At least two activities of the MTase were recognized; i.e., guanine N7 methyltransferase and nucleoside 2′-*O* ribose methyltransferase^16^. No MTase domain was found in the other genera of the *Flaviviridae* family, i.e., hepaciviruses and pestiviruses, which contain an internal ribosome entry site at its 5′-end of viral genome instead of the type 1 cap^17^.

The polymerase domain of NS5′ catalyses *de novo* RNA synthesis to first generate −RNA (negative-sense RNA) using the viral +RNA as a template. In the next round, the −RNA serves as a template for the synthesis of more +RNA strands which are used as mRNA for viral polyprotein synthesis or are packaged into arising viral particles^12^. NS5 is considered to be one of the most promising targets for antiviral drugs because both domains (MTase and RdRp) are essential for virus replication and are not present in non-infected cells^18–22^.

Recently, the structural biology of ZIKV RdRp significantly advanced as several crystal structures of the RdRp were analysed^23,24^ in addition to the previously solved structure by AbbVie Inc^15^. Additionally, several crystal structures of other flavivirus polymerases were solved in complex with small molecules^25,26^, however, neither of these structures contained an RNA molecule nor any nucleotide or nucleotide analog, which hinders their use in structure based design of novel inhibitors. For these reasons we endeavoured to build a sophisticated model of RNA and nucleotide bound ZIKV RdRp based on the published crystal structures of ZIKV RdRp and its most similar RdRp bound to RNA that can be found in the PDB database which is the Hepatitis C virus (HCV) RdRp. Such a functional model of ZIKV RdRp containing all the necessary components is indispensable for successful docking experiments, which are an essential part of the rational design of inhibitors that might serve as drugs against ZIKV and other flaviviruses. Correctness of the ZIKV RdRp:RNA structural model developed in this work is demonstrated biochemically using point mutants predicted from the structural model and the usefulness of our model is documented using docking experiments.

## Material and Methods

### Structural Model

**Figure 1:**
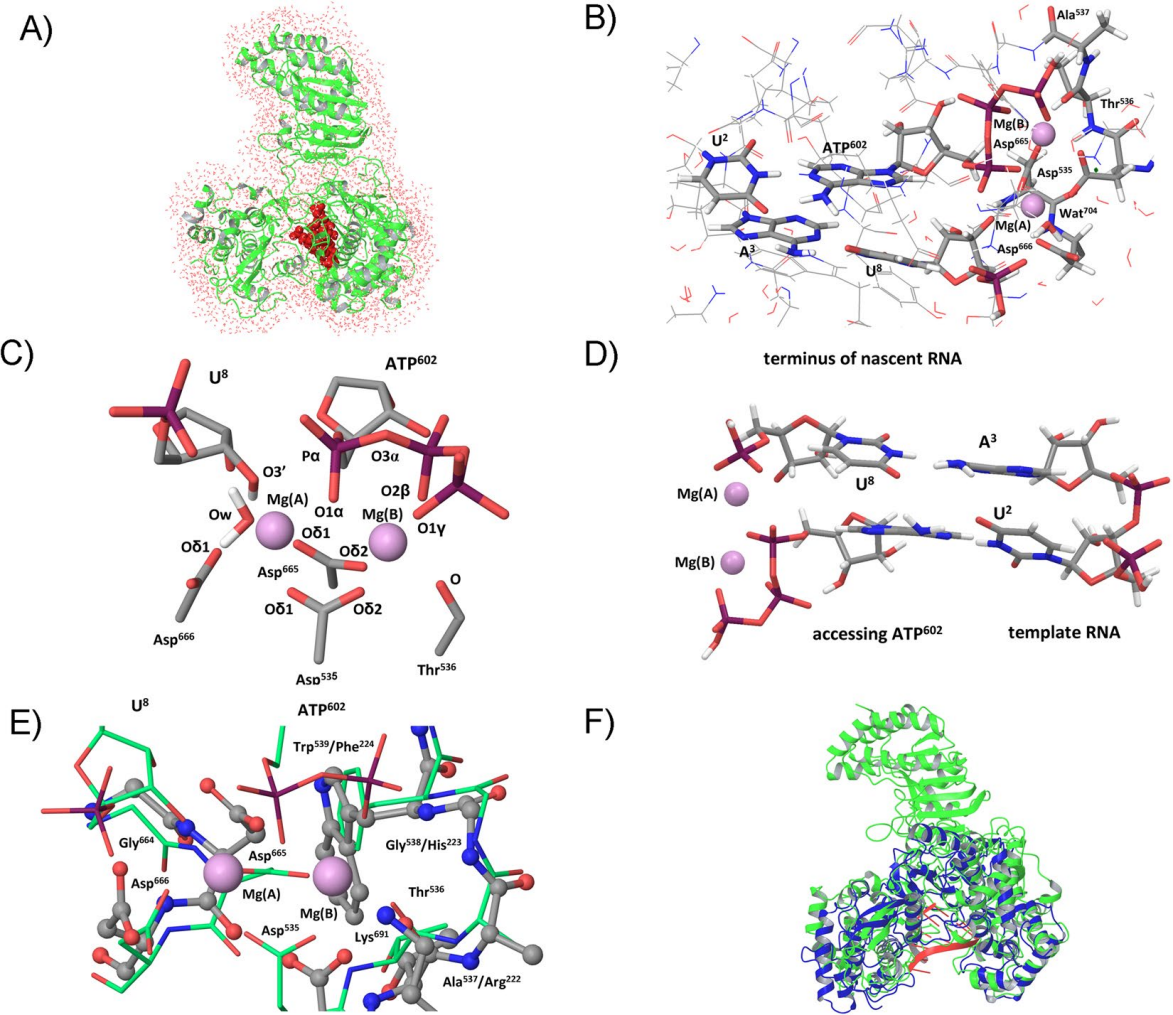
The ZIKV RdRp – RNA complex. (**A**) Sketch of the solvated structural model derived using the x-ray structures of HCV and Zika RdRps that was employed in QM/MM calculations: ZIKV (green), RNA (red), water molecules (red dots) (**B**) Sketch of the QM part (highlighted in bold) within structural model that included ATP^602^ and U^8^ nucleotides, U^2^ and A^3^ bases of paired nucleotides within template RNA, amino acids Asp^665^, Asp^666^, Asp^535^, The^536^, Ala^537^, two Mg^2+^ ions and the x-ray resolved water ligand of Mg atom and Wat^704^. The numbering of RNA was adopted from HCV 4WTD structure. The numbering of the amino acids was adopted from the ZIKV RdRp crystal structure 5TFR (**C**) The schematic sketch of the residues within catalytic core including their numbering used throughout the text. The balls Mg(A) and Mg(B) represent metal cations. (**D**) Sketch of the RNA molecules within catalytic core including two Mg atoms. (**E**) The alignment of catalytic core of HCV (thin-tube depiction including green carbon) and ZIKV RdRp (ball & stick depiction including gray carbon) structures obtained using the Chimera. The Gly^538^/His^223^, Trp^539^/Phe^224^, and Ala^537^/Arg^222^ denote the amino acid residues in ZIKV/HCV, respectively. (**F**) The overlay of ZIKV (green) and HCV (blue) inclusive RNA molecule (red) obtained using the Chimera program.

The QM/MM structural model was derived from the ZIKV RdRp (PDB ID 5TFR)^15^ and HCV (PDB ID 4WTD)^27^ crystal structures. The protein without substrate RNA was captured in 5TFR whereas the template RNA, nascent RNA and accessing ADP nucleotide within catalytic site were captured in 4WTD. The *S*-adenosylhomocysteine cofactor, bound to the MTase domain, was removed from the ZIKV structure. The structural alignment of ZIKV and HCV (chain B) performed using the Measures of Structural Similarity algorithm^28^ implemented in the Chimera^29^ yielded our starting structural model. The alignment of HCV and ZIKV RdRp involved 174 amino acid pairs and the RMSD between 174 pruned atom pairs was 1.123 Å (Figures S1 and S2). The two Mn^2+^ ions within the HCV catalytic core including their x-ray-resolved water ligands (two water molecules) were included in the structural model but were replaced by Mg^2+^ atoms because magnesium is the physiological ligand. The starting structural model thus corresponded to ZIKV 5TFR structure where the RNA molecules plus the x-ray resolved solvent within the catalytic core were adopted from HCV 4WTD structure. The ADP, which is the accessing RNA nucleotide in HCV, was in our structural model modified to ATP by adding a protonated γ-phosphate. The geometry of added γ-phosphate was adopted from the geometry of ATP in the x-ray structure of human norovirus polymerase (PDB ID 3H5Y)^30^. One of the two x-ray-resolved water molecules was removed because it occupied the position of the added γ-phosphate. The template RNA and nascent RNA strands were shortened to include only the last A^3^-U^8^ and consecutive nascent U^2^…A^602^ base pairs, where the A^602^ was accessing ATP (Fig. 1). The numbering of RNA nucleotides was adopted from HCV 4WTD structure and used throughout the text. The ZIKV RdRp complex was protonated assuming pH 7.0 using the Propka method^31^ and the Maestro program^32^.

The initial structural model was solvated to describe the effect on QM/MM-optimized geometries consistently because only one water ligand of the metal cation was crystallographically resolved within the catalytic site (Figs 1A and S3). Moreover, two alternative structural models were prepared assuming a different protonation state of the O3′ oxygen atom in nucleotide U^8^ that terminated the nascent RNA strand. One model included O3′-protonated ribose while the other included O3′-deprotonated ribose of U^8^. Geometries of the two structural models were preliminarily QM/MM-optimized and the complexes were solvated with the SPC explicit water^33^ using the Maestro program. A water layer with a thickness ~10 Å surrounding the ZIKV-RNA complex included 32 354 water molecules. A neutral charge of the solvated complexes was achieved by adding Cl^−^ anions using the Maestro program. The geometries of the protein, RNAs, two Mg^2+^ ions and the x-ray-resolved water within catalytic core were constrained during MD simulation by imposing the force constant 100.0 kcal mol^−1^ Å^−1^. Hence, only dynamics of the added water molecules and counter-ions was MD-calculated freely, without restraints to encompass the ZIKV-RNA complex with the explicit solvent coherently. The MD simulations were carried out by employing the Desmod program^34^. The OPLS-AA(2005) force field was used in all MD simulation^35^. First, the system was equilibrated employing the constraints and periodic boundary conditions, T = 300 K and P = 101325 Pa. The equilibration was followed by 120 ps of NVT simulation. The solvation of ZIKV-RNA complex was obtained upon 120 ps NPT MD simulation. The solvated structural model used in QM/MM calculations included only the water molecules within the 5 Å layer surrounding the ZIKV RdRp:RNA complex (Fig. 1A). The structural model of the protein-RNA complex was assembled by means of the two x-ray structures. When the interfacial parts of merged moieties are only loosely defined, e.g. in weak and transient complexes, their mutual arrangements should be extensively MD-sampled^36^. By contrast, the catalytic core of Zika-RNA can be regarded structurally well-defined and conserved within the family of polymerases^37^. The MD approach was, therefore, used only for relaxation of the initial complex while the QM/MM modelling approach ensured accurate optimizations of the catalytic core including key protein-RNA interactions in response to particular RNA sequences. Lastly, the structural models including all possible nascent RNA base pairs were prepared. For the structural model including O3′-deprotonated ribose of U^8^ four structural models included A^602^-U^2^, U^602^-A^2^, G^602^-C^2^ and C^602^-G^2^ base pairs. The structural model including normal U^8^ included merely A^602^-U^2^. The PDB files including respective five structural models can be found in the Supporting Information.

### The QM/MM Calculation

The QM/MM method was used for optimization of geometry of the ZIKV-RNA complex. The QM and MM part of structural model was described employing the QSite 6.1 program. The QM part included nucleotide-triphosphate N^602^, N=A, U, G, C, nucleotide U^8^, bases of the paired nucleotides within template RNA, amino acids Asp^665^, Asp^666^, Asp^535^, The^536^, Ala^537^, two Mg^2+^ ions and the x-ray resolved water ligand of Mg (Fig. 1B). The numbering of the amino acids adopted from 5TFR was used throughout the text. The charge of the QM part was −3 e^−^ and −4 e^−^ for structural model including O3′-deprotonated U^8^ or A^8^ and normal U^8^ nucleotide, respectively. The total charge of QM/MM model that included O3′-deprotonated U^8^ or A^8^ was 6 e^−^. The total charge of the model with normal U^8^ was 8 e^−^. The QM part was calculated using the DFT method employing the PBE functional^38^ and the lacvp* basis^39^. The MM part was calculated using the OPLS-AA(2005) force field^35^. Only the x-ray geometry of the sugar-backbone part of the template RNA was fixed in all QM/MM geometry optimizations, the rest of the geometric parameters was optimized. All the QM/MM-calculated energy minima were validated with the vibration analysis employing the latest Hessian as implemented within the QSite program^40^. Note, actual accuracy of the calculated structures de facto corresponds to the accuracies of original x-ray structures employed in preparation of initial Zika-RNA model. Somewhat higher precision of geometrical parameters throughout the text, however, corresponds to the accuracy assumed for the QM/MM method.

### Protein expression and purification

Wild type ZIKV RdRp and all the six mutants were expressed in *E. coli* Rosetta Gami B (DE3) cells and purified as described in detail previously^20^ using our using our standard protocols^41,42^. Briefly, the ZIKV RdRp (MR766 strain, residues 35–903 which corresponds to residues 2551–3419 of the polyprotein) was expressed with a His_8_SUMO purification and solubilisation tag. Bacterial lysate was prepared using French pressure cell press (Thermo). Proteins were purified by combination of Ni-NTA chelation chromatography followed by the cleavage of the purification tag by the Ulp1 protease and finally purified using size exclusion chromatography (SEC). The purified protein was concentrated to 10 μM concentration and stored at −80 °C in SEC buffer (500 mM NaCl, 20 mM Tris pH 8, 3 mM βME, 5% glycerol, 2 mM MgCl_2_) until needed.

### Enzymatic activity assays

The activity of ZIKV RdRp was measured as described previously^20^. Briefly, it was measured as a frequency of radioactivity labeled ATP* (alpha – P32) incorporation into prolonging chain. We used 0.2 ng/μl poly-U (Polyuridylic acid homopolymer, Sigma) as a template, 10 nM 15-mer poly-A primer and 1 μM ZIKV RdRp in buffer (50 mM Tris-HCl pH 7.5, 40 mM NaCl, 1 mM DTT, 2.5 mM MgCl2, 0.1 mg/ml BSA and 0.2 U/μl RNase inhibitor, Promega). The reaction was started by addition of 2.5 μM ATP and 3000 Ci/mmol ATP* (alpha -P32) and was running for 40 minutes at 30 °C. The RNA product was caught on anion exchange paper (Whatman, UK). Unbound radioactivity was washed out 10x with 125 mM Na_2_HPO_4_ (pH 9), twice with water and once with an ethanol flush. The radiation was measured by a Phosphorimager (Typhoon 9410) and the signal was quantified using ImageJ software^43^. Reactions without RdRp were used as a negative control and its signal was subtracted from all testing signals. The normalized activity was calculated as a ratio of wild type RdRp (100% activity).

### Docking

#### Protein preparation

The QM/MM-optimized structural model including ATP (au_o3.pdb) was used for all the docking experiments. Initially, the ATP as well as water molecules were removed. The pdbqt file was prepared with the AutoDock Tools 1.5.6^44^ using the default methodology (the polar hydrogen atoms were maintained in the model). The charges of Mg^2+^ ions were calculated for the QM part of QM/MM-optimized protein-RNA complex that included ATP^602^ and U^8^ nascent RNA base pair excluding the A^3^ and U^2^ nucleobases. The four hydrogen caps bridging the QM part with the MM part were replaced by hydrogen atoms that were geometry optimized with the PBE method and lapcv* basis using the Jaguar 8.2 program^45^. The RESP^46^ charges of the magnesium atoms were calculated using the B3LYP^47,48^ method and 6–31 G(d,p)^49,50^ basis set with the Gaussian09.D01^51^ quantum chemistry package and Amber tool antechamber^52^. The charge +1.1 e^−^ was calculated for the two magnesium atoms by using both alternatives of the structural models including normal and O3′-deprotonated ribose of U^8^.

The QM-calculated charges of the magnesium ions were manually inserted into the pdbqt file. We also extracted several water molecules (number 10, 15, 5056, 5594 and 5382 in our supplementary model files), which play an essential role in the binding site, from the original pdb file into a separate pdb file, prepared pdbqt file containing these water molecules in AutoDock Tool 1.5.6 and manually inserted them back into the pdbqt file with protein and ions.

#### Ligand preparation

ATP was extracted from the original model into a separate pdb file and converted to pdbqt by AutoDock Tools 1.5.6. We used ACD/ChemSketch 12.01 for *de novo* preparation of the ligand NIT008 and converted the 2D structure to a 3D structure using standard function with the force field based on CHARMM parametrization. The 2′-endo (South) and 3′-endo (North) conformations of this compound were prepared and optimized using B3LYP-D3^53^ method and 6–31(d,p) basis set with inclusion of the implicit water environment modelled by Polarizable Continuum Model (PCM)^54^. The necessary conversion of the format was performed using AutoDock Tools 1.5.6.

#### Docking validation and docking experiments

We have performed several validation experiments, which show the importance of all the components (RNA, nucleotide, RdRp) for successful docking experiments. All these experiments were performed using Autodock Vina 1.1^55^ with the default docking method at larger exhaustiveness = 200 and greater binding space (binding pocket of 30 × 30 × 30 Å centered at −2.55, −7.53 and 1.67 Å) in comparison with the subsequent docking experiments. The docking experiments were also performed with the Autodock Vina 1.1 using the default docking method with exhaustiveness = 100 under similar conditions as in our previous work^56,57^. The docking was performed into the smaller binding pocket of 20 × 20 × 20 Å centred at −2.55, −7.53 and 1.67 Å.

## Results

### The QM/MM calculations of ZIKV RdRp:RNA complex

The ZIKV RdRp:RNA model employed in hybrid quantum mechanics/molecular mechanics (QM/MM) calculations was derived from the ZIKV RdRp (PDB ID 5TFR) and HCV (PDB ID 4WTD) crystal structures because no flaviviral RdRp was so far crystallized in complex with RNA. The two structures were superimposed using the Measures of Structural Similarity algorithm^28^ as implemented in the Chimera^58^. The alignment of HCV and ZIKV RdRp involved 174 amino acid pairs and the RMSD between 174 pruned atom pairs was 1.123 Å (Figures S1 and S2). Next, the RNA and x-ray-resolved solvent in the HCV structure were transposed into the ZIKV structure and the two Mn^2+^ ions atoms were replaced by the physiological Mg^2+^ ions. Finally, the ADP from HCV was modified to ATP as detailed in the M&M section. The RNA strands were shortened to include only the A^3^-U^8^ and nascent U^2^…A^602^ base pairs (the numbering of residues was adopted from the HCV RdRp structure 4WTD). The protein-RNA complex was protonated assuming pH = 7.0 and solvated by explicit water molecules keeping all geometry parameters derived from the two crystal structures fixed as is summarized in Fig. 1. Two alternative structural models, one including normal ribose of U8 (Fig. 1C, supplementary PDB file au_o3h) and one included O3′-deprotonated ribose of U^8^ (Fig. 1D, supplementary PDB files au_o3′, cg_o3′, gc_o3′, ua_o3′) describe the two possible states of the principal catalytic nucleotide. The solvated structural models furthermore included four possible nascent RNA base pairs to model the catalytic core including all the four NTP^602^, N=A, C, G, U.

We discovered that the ordering of the RNA molecules within catalytic site indicated the A-form of the nascent RNA albeit local geometries of nucleotides and their mutual arrangements did not correspond precisely to the canonical A-RNA. At first, the class of backbone of the template RNA was not the A-form although the majority of the backbone torsions fit the A-RNA values (Table S1). Secondly, the bases within base pairs did not lie in one plane. Interestingly, the starting crystal structure was more of a buckle-type of mutual declination (Figure S4) while the deviations from their ideally planar arrangements within QM/MM-optimized base pairs was more likely a propeller-twist (Figures S5–S9). In any case, the mutual declination of bases in base pairs was noticeable. The propeller-twist in QM/MM-optimized base pairs ranged from ca 20° to 30° and in the U^602^-A^2^ pair it was even 41° (Table S4). The buckle angle of the base-planes in the case of the HCV polymerase bound to RNA (pdb code 4WTD) was far smaller (Table S4). This incoherence is most likely due to no geometry restraints in the QM/MM method compared to the crystallographic refinement employing planarity geometric restrains for the nucleobases in structures with a resolution worse than 1 Å (The original crystal structure 4WTD was solved at 2.7 Å resolution.). Therefore, the out-of-plane deviations of the inner-ring atoms of the nucleobases within DNA and RNA molecules can be observed only in crystal structures obtained at ultra-high resolution (1.0 Å or better) that are refined without geometry based restrains^59^. Just minutely disordered geometries of the RNA nucleotides in the RdRp:RNA complex as compared to canonical A-RNA inevitably resulted in slightly imperfect geometries of the nascent base pairs (Figure S4). However, the base pairing pattern adopted from the catalytic core of the HCV polymerase was sustained during all QM/MM calculations (Figures S5–S9), which illustrated considerable versatility of the catalytic site with respect to all possible sequences of captured RNAs. The hydrogen bonds within the canonical Watson-Crick base pairs can be derived from the respective interatomic distances between the H-bonded atoms in the crystal structure (Figure S4) and in all QM/MM-calculated geometries (Figures S5–S9). The QM/MM geometry optimization resulted in rather directional (linear) character of the hydrogen bonds within the base pairs, however, at the expense of their notable propeller twist. In any case, both the X-ray and QM/MM geometries of base pairs illustrated rather notable deviation of the RNA strands within ZIKV RdRp catalytic core from the ideal A-form RNA duplex.

Interestingly, the extent of mutual declination of the bases in the base pairs depended on the RNA sequence (Table S4). Probably the most striking was the difference of propeller-twist angles 21° and 8° calculated in U^8^-A^3^ and A^8^-U^3^ base pair, respectively. A closer look into the two base pairs unveiled that notable out-of-plane geometries of the paired bases pairs allowed RNA sequence-specific interactions that would not be possible in canonical A-RNA (Figure S5). The inter-base pair interactions involving NH_2_ and CO groups of bases were possible due to the imperfect geometries of nascent RNA duplexes and particularly due to their sequence that involved combinations of suitable stacked purine-pyrimidine bases (e.g. the upper structure in Figure S5). These interactions would be impossible in the case of the stacked purine-purine and/or pyrimidine-pyrimidine bases (e.g. the bottom structure in Figure S5). Lack of the NH_2_…O=C inter-base interactions between homologous stacked bases likely explains the smaller propeller-twist of A^8^-U^3^ relative to that of U^8^-A^3^ (Figure S5). Though locally different, the overall QM/MM-calculated geometries of RNA nucleotides corresponded to the x-ray geometry of the HCV catalytic core, which represents a reliable initial state of the catalytic reaction where the accessing nucleotide is going to be connected to the growing nascent RNA strand according to the template RNA (Fig. 1D).

The QM/MM-calculated geometries of RNA molecules within the catalytic site illustrated some aspects that could be important with respect to the function of the Zika polymerase. Notably, the geometries of the two Mg^2+^ cations coordinated to both RNA and protein revealed their putative function. Close proximity of the O3′ oxygen of terminal nucleotide 8 to Mg^2+^(A) cation and to the Pα atom of the NTP (Fig. 1C) indicates activation of the terminal RNA nucleotide within the nascent RNA strand that facilitates formation of the O3′-Pα phosphodiester linkage. The QM/MM-calculated O3′-Mg^2+^(A) distance depended critically on whether the terminal nucleotide 8 of nascent RNA was or was not O3′-deprotonated (Table S3) while the dependence on RNA sequence was negligible (Table S2). Noteworthy, the metal-ligand distances calculated for Mg^2+^ and Mn^2+^ differed (Table S2), which may explain the metal-specific effect on catalytic function that was reported for the HCV RdRp^60^. For O3′H-form of terminal RNA nucleotide 8 the calculated O3′-Mg(A) and O3′-Mn(A) distance was 2.194 Å and 2.280 Å, respectively. The O3′-Mn(A) distance in the HCV RdRp crystal structure was 2.129 Å. For O3′-deprotonated nucleotide 8, the O3′-Mg(A) distance ranged from 1.970 Å to 1.977 Å (Table S2) and the O3′-Mn(A) distance was 1.989 Å (Table S3). The QM/MM calculations thus indicated that the O3′H-form of U^8^ was captured in the HCV RdRp crystal structure. The activation of the RNA molecules with regard to the catalytic reaction due to O3′-deprotonation of terminal nucleotide of nascent RNA was further demonstrated by shortening the distance between the O3′ of nucleotide 8 and Pα of NTP^602^. For ATP, the O3′ nucleotide 8-Pα shortened upon O3′-deprotonation from 3.142 Å to 2. 926 Å. For the remaining three NTPs and O3′-deprotonated ribose of nucleotide 8, the O3′-Pα ranged from 2.892 Å to 2.929 Å (Table S5). The O3′-Pα in HCV crystal structure was 3.366 Å, which again indicates that the prevalent, normal form of the terminal RNA nucleotide 8 was captured in the HCV RdRp crystal. The O3′-Pα calculated with Mn^2+^ for normal and O3′-deprotonated ribose of nucleotide 8 was 3.268 Å and 3.076 Å, respectively. The activated state of the RNA substrate, however, assumes O3′-deprotonation of the terminal nucleoside within the nascent RNA that takes place in the catalytic core^61^. Consequently, the majority of the QM/MM calculations employed O3′-deprotonated nucleotide 8 to illustrate the reaction state shortly before formation of the O3′-Pα phosphodiester bond.

### Biochemical validation of the ZIKV RdRp:RNA complex model

**Figure 2:**
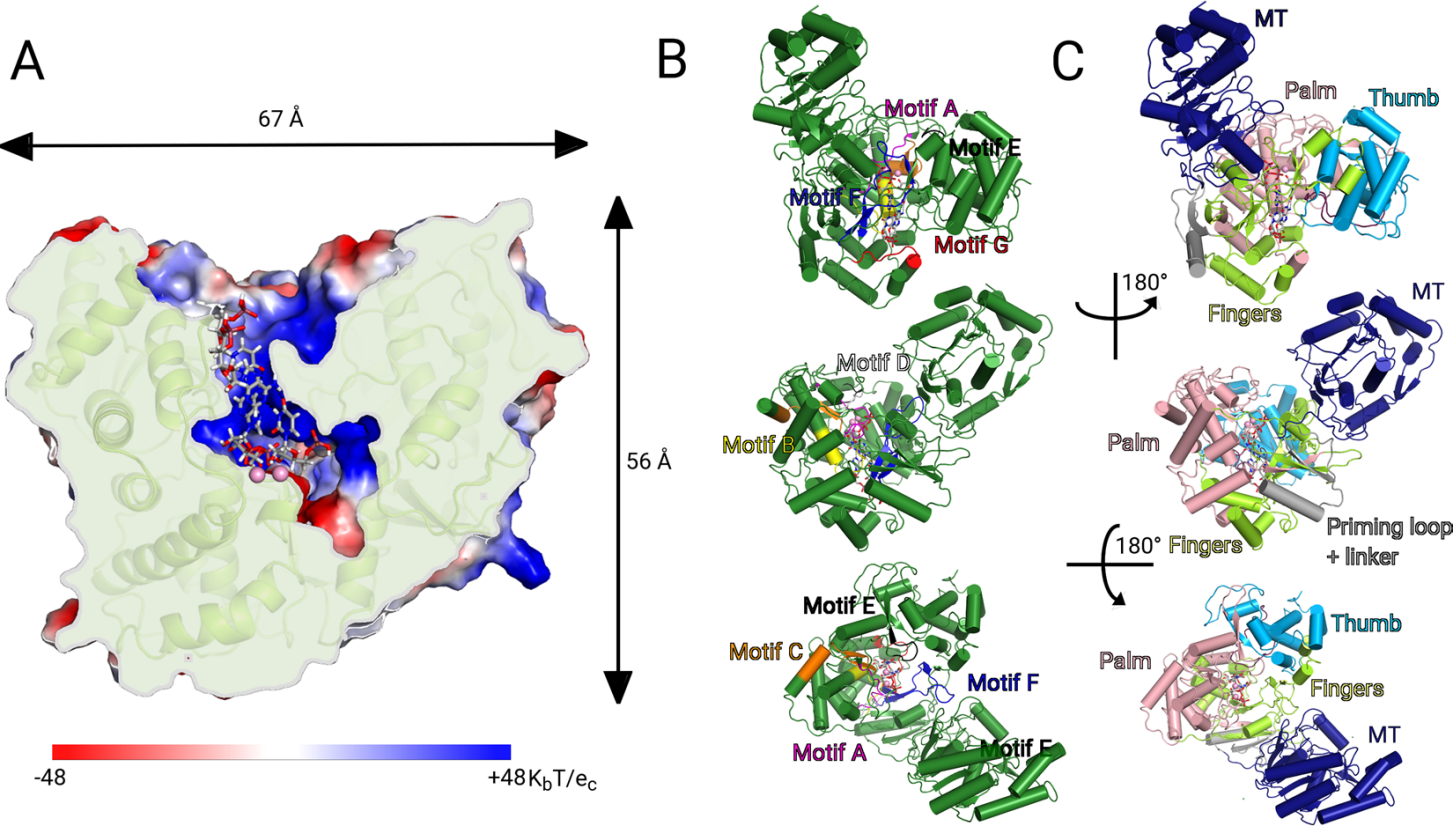
ZIKV RdRp model – biological significance. (**A**) The cross-section of ZIKV RdRp, the electrostatic surface is shown in red – blue conversion. Positively charged areas are blue and negatively charged areas are red. The RNA binding cavity in the protein catalytic core has positive charge that is complementary to the negatively charged RNA. (**B**) Structural motifs representations. Motif A (pink), Motif B (lime), Motif C (orange), Motif D (white), Motif E (black), Motif F (blue), Motif G (red) are highlighted on the ZIKV RdRp model. (**C**) Domains representations. On ZIKV RdRp can be distinguish 4 domains: Methyltransferase – MT (blue), Palm (pink), Fingers (green) and Thumb (cyan).

**Figure 3:**
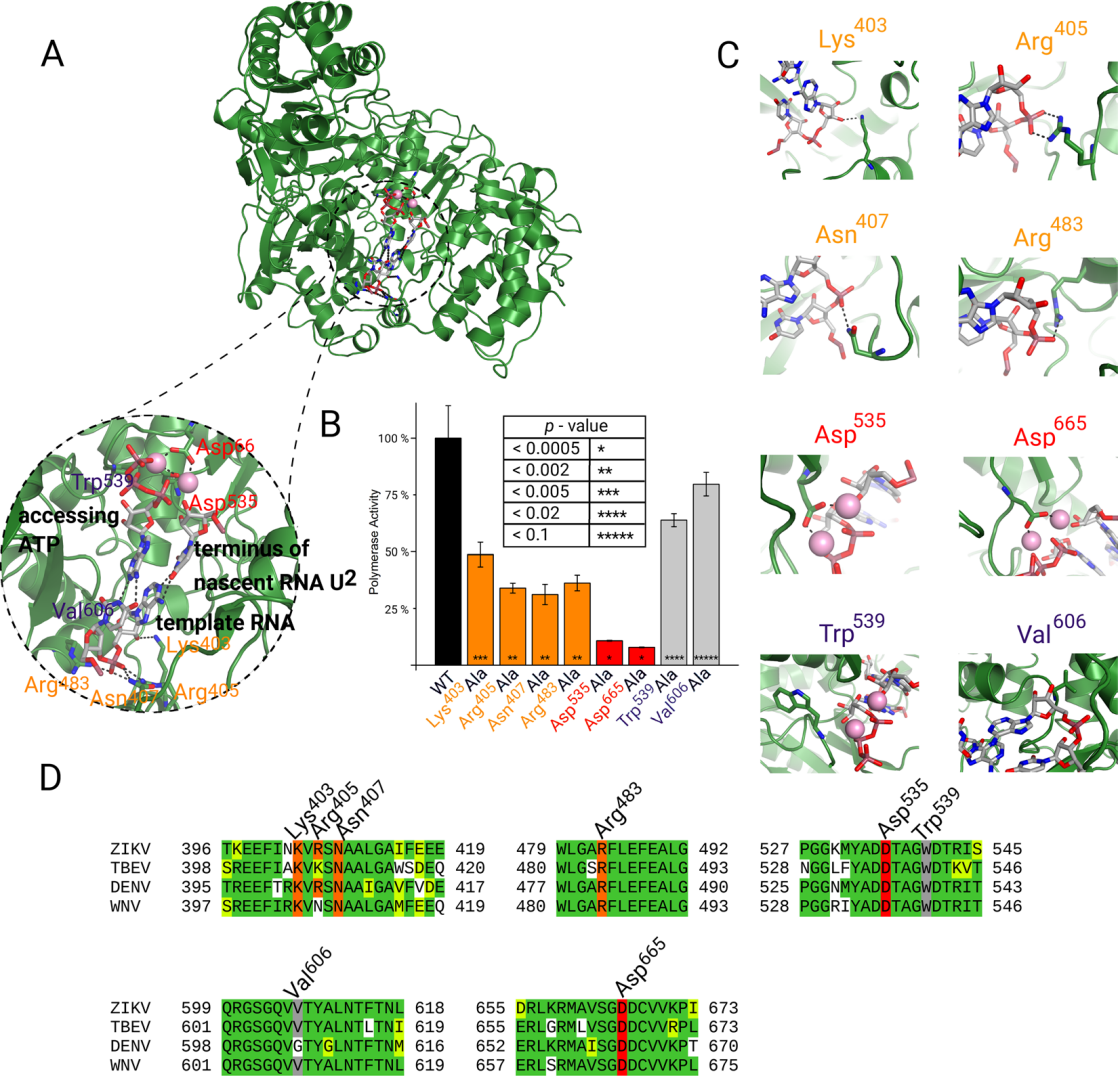
Enzymatic analysis. (**A**) ZIKV RdRp:RNA complex model with an enlarged RNA binding cavity. The RNA template is a dinucleotide composed of an A^3^ – U^8^ base pair. Nascent U^2^ and ATP are present. In enlarged picture are highlighted amino acids related to activity assay. (**B**) Activity of ZIKV RdRp: Mutations Lys^403^Ala, Arg^405^Ala, Asn^407^Ala, Arg^483^Ala significantly decrease activity (orange), whereas mutation Asp^535^Ala or Asp^665^Ala (red) totally disrupts RdRp enzymatic activity. In mutations Trp^539^Ala and Val^606^Ala (gray) were not achieved any significant difference. Values in box are Student t-test *p* values. (**C**) Detail of mentioned AAs – ligand wt ZIKV RdRp interaction: Lys^403^, Arg^405^, Asn^407^ and Arg^483^ provide interactions with template RNA, Asp^535^ and Asp^665^ provide interaction with Mg2+, Trp^539^ and Val^606^ are in the proximity of active site RdRp. (**D**) Amino acids sequence alignment RdRp through Flaviviruses: Zika virus 1 – ZIKV, Tick-borne encephalitis virus Hypr - TBEV, Dengue Virus 1– DENV, West Nile virus – WNV.

We chose six AAs playing key roles in RNA replication based on our model of the ZIKV RdRp:RNA complex (Fig. 2). Using PyMol (The PyMOL Molecular Graphics System, Version 1.8 Schrödinger, LLC) software, we identified four AAs (Lys^369^, Arg^371^, Asn^373^ and Arg^449^, orange color code in Fig. 3) that our model predicts to be important because they create H – bonds with RNA at a maximum distance of 3.2 Å. We also chose two AAs interacting with Mg^2+^ ions (Asp^501^ and Asp^631^) that are thus predicted essential (red color code in Fig. 3). As a negative control we used Trp^505^ and Val^572^ (grey color code in Fig. 3) that are located in the proximity of RNA, however, their interactions with RNA or Mg^2+^ are not essential on our model (Fig. 3). Next, we prepared individual recombinant ZIKV RdRps each bearing a single mutation in aforementioned AAs (mutation to Ala).

The activity of all these recombinant RdRp was measured as the ability to incorporate ATP* (alpha – P32) into elongated polyA RNA and compared with the wild type ZIKV RdRp (Fig. 3B). The ZIKV RdRp mutants involving AAs that create H – bond(s) with RNA (Lys^369^Ala, Arg^371^Ala, Asn^373^Ala, and Arg^449^Ala) had significantly lower activity compared to the wild type protein (decrease of 65%). Total loss of enzymatic activity was measured with the mutants involving AAs that interact with Mg^2+^ (Asp^501^ or Asp^631^mutated to alanine). The enzymatic activity of RdRp with mutation Trp^501^Ala was also reduced (40% reduction). Tryptophan is a large aromatic amino acid and although Trp^505^ does not directly participate in RNA polymerization (based on the model) it might be important for the stability of ZIKV RdRp. As was assumed, the mutation Val^572^Ala in ZIKV RdRp had negligible effect on the polymerase activity.

### Docking validation and docking experiments

The docking experiments and validation were performed by the open-source software AutoDock Vina 1.1 using the default methodology^55^. Water molecules were omitted for the docking experiments except for the water molecules within the active site.

**Figure 4:**
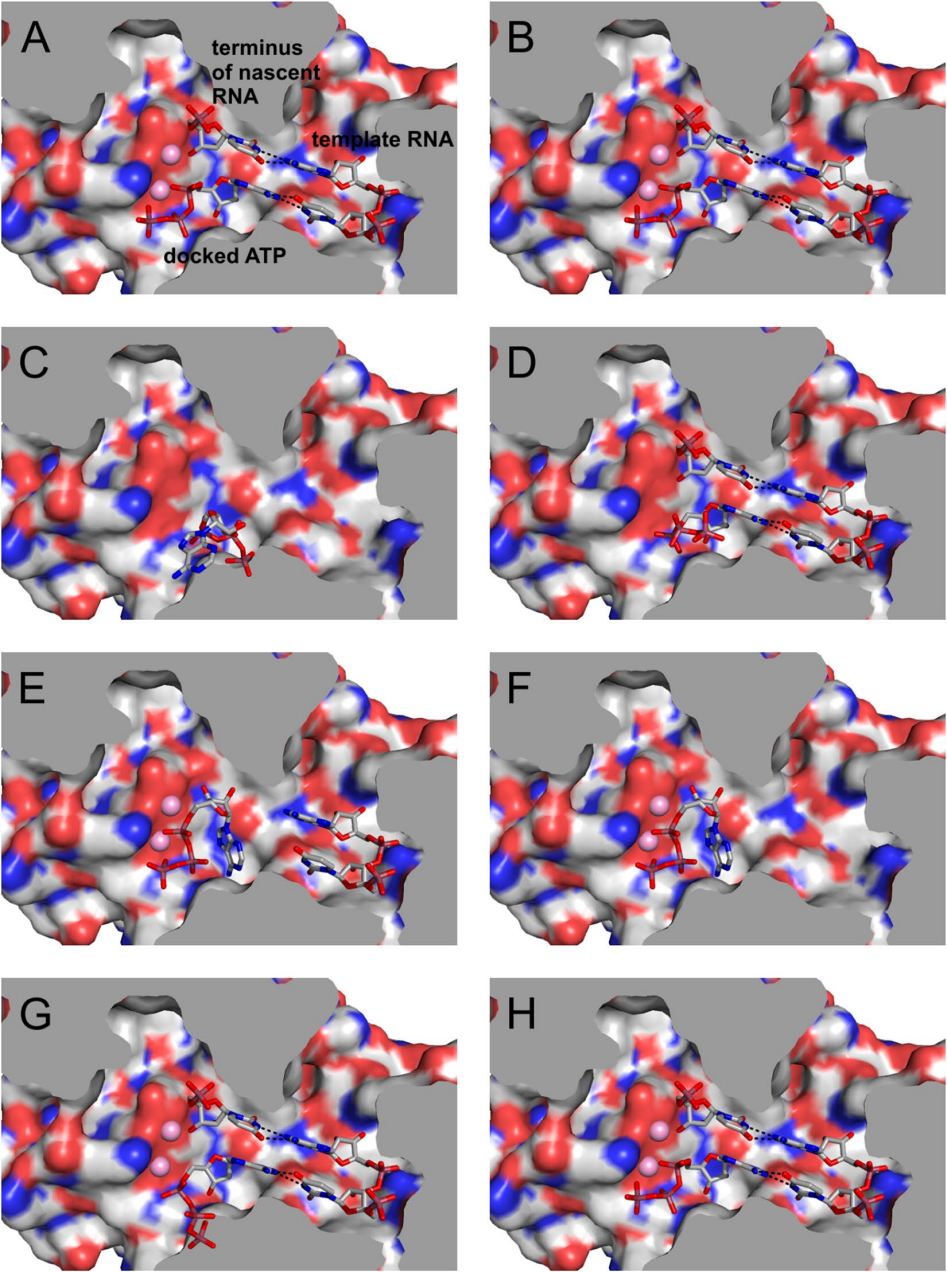
Results of the docking validation. (**A**) Docking of ATP using the standard procedure. (**B**) As in A but using expanded grid-box (30 × 30 × 30 Å). (**C**) Docking into the structure of Zika RdRp without any ligands (**D**) As in C but only magnesium ions and waters in the active site are omitted. (**E**) As in C but only the terminus of the nascent RNA was omitted. (**F**) As in C but only both RNA components were omitted. (**G**) As in A but the with mutated Arg^473^. (**H**) As in A but with mutated Lys^458^.

**Figure 5:**
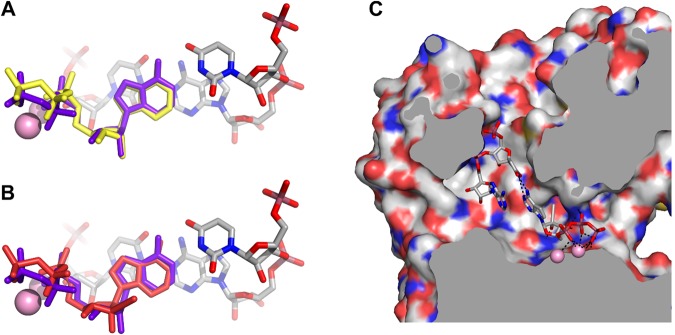
Results of the docking experiments. (**A**) Control re-docking of ATP into the model of ZIKV RNA-dependent RNA polymerase. The overlay of ATP from the original model (purple) and the structure docked by AutoDock Vina (yellow). The docking pose with the highest score is shown. (**B**,**C**) Docking of compound NITD008. (**B**) Overlay of the original ATP in the model (purple) and the highest score pose of NITD008 with *anti* orientation of the nucleobase (red). (**C**) The docking pose of NITD008 reveals significant lipophilic cavity occupied by the large 2′-ethynyl substituent.

Initially, we have performed several experiments to validate the importance of the distinct components of the presented model. Firstly, we have successfully tested this approach by the redocking of ATP into the model using two different grid-box sizes. Both these experimental setups resulted in similar results (Tables S6 and S7) that are summarized in the Fig. 4. Congruently, as also shown in Fig. 5A and Figure S11, the pose of re-docked ATP nicely correlates with its original position within the binding site of the RdRp that was QM/MM-optimized. In the second part of the validation process, we have gradually removed the parts of the model in the active site and evaluated the effect on docking. Docking of ATP into the model missing all the implemented components (nascent and template RNA, Mg^2+^ and waters) resulted in completely incorrect pose of ATP in the binding site (Fig. 4C). Docking into a model with removed Mg^2+^ ions and water molecules gave ATP with significant distortion of the triphosphate moiety but maintained interaction with the nascent and template RNA (Fig. 4D). In contrast, when the model lacks either nascent RNA strand or both RNA components, the docking fails due to distortion of the nucleoside moiety (Fig. 4E,F). In the validation step we have also mutated Arg^473^ and Lys^458^ to glycine. This experiments show that while the removal of Arg^473^ has a significant effect on the position of the triphosphate moiety of the docked ATP (Fig. 4G), the mutated Lys^458^ has none or only marginal effect on the docking results (Fig. 4H).

Subsequently, we used this validated model for docking of the well-known flavivirus RdRp inhibitor NITD008 (in its triphosphate form, Figure S10), which exerts significant activity against ZIKV and other flaviviruses^62,63^. We selected this model compound over numerous other 2′-substituted nucleoside derivatives, since compound NITD008 possess significantly sterically demanding ethynyl substituent at 2′-position. This ethynyl substituent requires significant space in the active site of the enzyme in order to be efficiently recognized. For the purpose of docking, we prepared two starting structures of NITD008 in two distinct conformations of the ribose ring – 2′-endo (South) and 3′-endo (North) because the docking algorithm employing AutoDock Vina does not alter the conformation of the ribose ring. The two conformers of NITD008 employed in the docking studies were geometry optimized using the B3LYP-D3^53^ method, 6–31(d,p) basis set and by including the effect of implicit water modelled by the Polarizable Continuum Model (PCM)^54^.

The results of NITD008 docking clearly show that only the 3′-endo (North) conformation (Fig. 5) fits nicely into the active site without any significant NITD008 distortion. As shown in Fig. 5B,C, the position of the NITD008 is similar to that of ATP and NITD008 occupies a lipophilic cavity of the RdRp protein adjacent to the 2′-position of the ribose moiety. Although the 2′-endo (South) conformer of the NITD008 derivative also directed the ethynyl substituent into this lipophilic cavity, the score calculated for this conformer was significantly lower (9.3 kcal over 10.1 kcal – Tables S14 and S15, Figures S12 and S13) than the score of the 3′-endo (North) counterpart.

## Discussion

The QM/MM-optimized model of the ZIKV RdRp:RNA complex highlighted many important interactions among all the partners within the catalytic core that involved Mg^2+^ ions, accessing NTP, template, and nascent RNAs and the protein. First, we will focus on the local geometric parameters to illustrate the structural basis for rationalizing the docking studies and the mutation experiments.

The geometries of the Mg^2+^ ions (Tables S2 and S3) were affected by RNA sequence less than the RNA-protein geometries (Table S5). This illustrates well-conserved structure of the very catalytic core near the key catalytic metal (A) (Fig. 1B) as compared to the rather versatile structure of RNA-protein interface that must be naturally adjusted according to RNA sequence. The lengthening of O3′α-Pα bond in ATP upon O3′-deprotonation of the terminal nucleotide of nascent RNA by 0.028 Å illustrates activation of the accessing NTP. The O3′-deprotonation of the terminal RNA nucleotide 8, at the same time, resulted in shortening of the coordination bond between the catalytic metal and O3′ oxygen of 8 that is favourable with respect to consequential formation of the O3′-Pα phosphodiester bond. The catalytic Mg^2+^ ion (A) clearly provides the principal linkage of the nascent RNA with the accessing NTP that is activated only upon O3′-deprotonation of the terminal nucleotide within the nascent RNA. This assumption is coherent with recent benchmark calculations of the P-O bond formation operated by DNA Polymerases β and λ^61^. Our QM/MM calculations furthermore revealed that the interactions between the NTP phosphate and other RNA molecules remained preserved, only respective bond distances varied upon altered RNA sequence (Table S5). These results clearly show versatility of the forthcoming catalytic reaction irrespective of the RNA sequence. By contrast, the interactions of RNA nucleobases with protein depended far more on RNA sequence, however, some interactions, such as the O2′^602^ – Nδ_2_ Asn^612^, O2′^8^ – N Asp^655^ and O3′^3^ – Nε Lys^403^, were preserved irrespectively of the RNA sequence (Table S5, section Base(RNA)-protein). Otherwise, variety of sequence-dependent changes occurred within the network of nucleobase-protein interactions. Firstly, the replacement of the nucleobases in the RNA molecules resulted in virtually the same interaction, only the contact was mediated by a different atom. For example, the Nε nitrogen of Lys^458^ interacted with N7 nitrogen of ATP^602^, O6 oxygen of GTP^602^ and N4 nitrogen of UTP^602^ and CTP^602^ in dependence of the RNA sequence (Table S5). Locally, the Nε of Lys^458^ established H-bond with N7 and O6 atoms of the purine and with the C4-amino group of the pyrimidine bases. These adjustments of the local interactions according to the atoms and groups of atoms within RNA nucleobase that occupy one, rather distinct position within catalytic core were also accompanied by a change of the interacting atom within the amino acid. For example, the C_β_, C_γ2_ or C_δ1_ carbon atoms of Ile^475^ amino acid were involved in highly sequence-dependent interactions with the nucleotides 2 and 602 (Table S5). The calculations of RNA-protein interactions illustrated versatility of the amino acid residues within the ZIKV RdRp catalytic site. Proper deposition of the RNA molecules within the catalytic core, indispensable with regard to the catalytic reaction, was shown to be maintained irrespective of the RNA sequence.

The QM/MM calculations also demonstrated a rather conservative pattern of the RNA phosphate-metal-protein interactions within ZIKV RdRp catalytic core as compared to somewhat more versatile nucleobase-protein interactions that were obviously adjusted according to the particular RNA sequence. Hence, the homologous parts of the RNA molecules involving particularly phosphate and ribose units are deposited within the ZIKV RdRp core in a uniform way regardless of RNA sequence namely due to the effect of the two catalytically important Mg^2+^ cations. Especially the Mg^2+^(A) was critical with respect to RNA activation as it provides principal chemical linkage of the nascent RNA strand with the accessing NTP. Highly variable nucleobase-protein interaction network demonstrated versatility of the amino acid residues within catalytic core that is indispensable for proper deposition and capturing of RNA molecules irrespectively of their sequence. The contacts among RNAs, the two Mg^2+^ cations and the ZIKV RdRp, which were geometrically characterized in Tables S2–S5 were employed for design of biochemical experiments for independent validation of our model.

ZIKV RdRp has a right - handed fold with palm, fingers, and thumb domains typical for all polymerases. Seven structural motifs can be distinguished throughout the RdRps^64^. Motifs A – E are included in the palm domain and motifs F – G in the fingers domains (Fig. 2B). Two universally conserved aspartic acids residues (Asp^535^ that is located in motif A and Asp^665^ is in motif C) are responsible for interaction with Mg^2+^ ions and mediate the two metal ion catalysis^65^ that enhances substrate recognition and catalytic specificity^66^. Mutation of either Asp^535^ or Asp^665^ results in a total loss of ZIKV RdRp polymerization activity (Fig. 3B). Motif G winds around the RNA template strand and is specific for RdRps. It contains the important residues Lys^403^, Arg^405^, Asn^407^ and Arg^483^. In ZIKV RdRp the Arg^405^, Asn^407^ and Arg^483^ residues are predicted to interact with the phosphodiester backbone of the template RNA. Mutation of any of these residues to Ala results in a significant loss of polymerization activity (Fig. 3B). Lys^403^ (localized in the beginning of motif G) is highly evolutionary conserved in tick-borne encephalitis virus (TBEV), Dengue virus (DENV) and ZIKV RdRps. Our model predicts that residue Lys^403^ interacts with ribose 3′-hydroxyl group of the template RNA (Fig. 3C). Not surprisingly, the replacement of Lys^403^ by Ala had a significant effect on polymerase activity in accordance with our prediction (Fig. 3B).

Motif B is located in the palm domain and contains Ser - Gly residues responsible for RNA trans-location. However, polymerases with mutated Ser residues retained their ability to incorporate multiple nucleotides^67^. Val^606^ is in close proximity to catalytically important Ser – Gly pair. However, our model predicted that Val^606^ is not important for the enzymatic activity of RdRp. In accordance, we observed only a slight decrease of polymerase activity in the Val^606^Ala mutant. Motif A participates in the coordination of two catalytic magnesium ions^68^ and contains Trp^559^ that is located close to the catalytic centre of ZIKV RdRp. Although our model predicted that Trp^559^ does not form any hydrogen bonds with the substrate we observed somewhat reduced enzymatic activity (~40%) in the Ala mutant. We therefore suggest that the large aromatic Trp^559^ residue is required for proper folding and/or stability of ZIKV RdRp. Alternatively, Trp^559^ could contribute to membrane binding as often aromatic residues do^69,70^ and viral RdRps are known to be membrane associated^71,72^, however, such an aromatic residue needs to be surface exposed and Trp^559^ is rather buried within close proximity of the active site.

The docking experiments, which we performed using our model with ATP as the accessing NTP, clearly showed that using simple docking software such as AutoDock Vina can lead to satisfactory results. Our experiments with the redocking of ATP resulted in a very nice overlay of the original ATP pose optimized by QM/MM calculations (Fig. 5A). The validation of the model proved that the inserted components (magnesium ions, RNA components and water molecules) play a vital role in docking and their removal leads to significantly incorrect docking results (Fig. 4C–H, Tables S8–S13) in comparison with the docking results obtained for QM/MM optimized model containing these essential parts of the polymerase reaction (Fig. 4A,B, Tables S6 and S7). Subsequently we demonstrated that the compound NITD008, the well-known inhibitor of flavivirus RdRp^73^, can be effectively docked into the binding site despite the bulky alkyne substituent at 2′-position.

Nonetheless, several parameters appear to be essential for successful docking. Firstly, the appropriate charge of the magnesium ions seems to be critical for the optimum positioning of the docked ligand, which is in agreement with the docking studies performed by Chen and co-workers^74^. The most favorable results were obtained when the formal charges of magnesium atoms were set to +1.1 e^−^. (This charge was QM calculated for both magnesium cations). Secondly, the MD calculated and QM/MM geometry optimized solvation of the active site of the RdRp seems to be crucial for formation of appropriate interaction(s) of the triphosphate with the magnesium ions and subsequent position of the nucleobase. However, omission of the water molecules adjacent to the nucleobase might be necessary for appropriate docking of the base modified nucleoside/tide derivatives. Finally, the experiment performed with NITD008 clearly shows that conformation of the ribose ring plays a crucial role in successful docking and it can significantly affect the results. Our data showed that NITD008 in 3′-endo (North) conformation can accommodate the binding site of ZIKV RdRp in an efficient way with minimum distortion of both the nucleobase and the sugar moieties in comparison to ATP in the original model (Fig. 5B). NITD008 in the 2′-endo (South) conformation also fits into the binding site with the alkyne moiety placed in the lipophilic cavity, however, the score was approximately 1 kcal lower as compared to the score of the North conformation. In addition, both the nucleobase and the triphosphate part were significantly disordered. These docking experiments indicated that NITD008 3′-endo (North) conformation is the active conformation against the ZIKV RdRd. It is, therefore, tempting to suggest that the preferred conformation depends on the individual properties of distinct nucleotides rather than being governed by some general rule.

## Conclusions

In conclusion, the comprehensive structural model of ZIKV RdRp:RNA including the protein, template and nascent RNAs, approaching nucleoside triphosphate and magnesium ions was derived from available crystal structures. The QM/MM-optimized ZIKV RdRp:RNA models including all possible nascent RNA base pairs are designated for future docking studies aimed at the design of novel nucleotide analogues against the ZIKV RdRp. These models will be also very useful for the rationalization of the biochemical experiments focused on the catalytic function of ZIKV RdRp because no structural model that includes both the protein and the RNAs is available at the moment. Exploitation of the ZIKV RdRp:RNA model was successfully demonstrated with the docking of NITD008, a nucleoside triphosphate analog. The protein-mutation study assuming the picture of catalytic core was rationalized by using our RdRp:RNA model. In particular, the classification of contacts among amino acids within the core, RNA and Mg^2+^ ions was employed in rationalization of measured catalytic function of the six mutants as compared to the activity of the wild type enzyme. The structural model highlighted the importance of particular amino acid residues in direct contact either with RNA molecules and/or magnesium ions. By contrast, mutation of the adjacent residues, which are not involved in the direct contact with these essential components of the reaction, exerted significantly lower effect on polymerase activity. The ZIKV RdRp:RNA models provide solid basis for molecular modelling of RdRp behaviour in relation with its biochemical function. Therefore, it can be utilized by medicinal chemists for the design of novel nucleoside and nucleotide derivatives as direct-acting antiviral agents against the ZIKV and other flaviviral pathogens.

## Electronic supplementary material

Supplementary Information
