# Supplementary material for: The structural model of Zika virus RNA-dependent RNA polymerase in complex with RNA for rational design of novel nucleotide inhibitors
Source: Sci Rep. 2018 Jul 24;8:11132. doi: 10.1038/s41598-018-29459-7 (PMC6057956; doi:10.1038/s41598-018-29459-7)
Supplement: Supplementary file 1 — Supplementary Information [file 41598_2018_29459_MOESM1_ESM.pdf]

Supplementary Data

The structural model of Zika virus RNA-dependent RNA polymerase in complex with RNA for rational design of novel nucleotide inhibitors

Jakub Šebera[1,‡], Anna Dubánková[1,‡], Vladimír Sychrovský[2], Daniel Ruzek[3], Evzen Boura[1,*], Radim Nencka[1*]

[1] Gilead Sciences Research Centre at IOCB Prague, Institute of Organic Chemistry and Biochemistry of the Czech Academy of Sciences

[2] Institute of Organic Chemistry and Biochemistry of the Czech Academy of Sciences

[3] Veterinary Research Institute, Hudcova 70, CZ-62100 Brno, and Institute of Parasitology, Biology Centre of the Czech Academy of Sciences, Branisovska 31, CZ-37005 Ceske Budejovice, Czech Republic

‡ These authors contributed equally.

* To whom correspondence should be addressed.

Radim Nencka

Tel: +420220183265

Email: nencka@uochb.cas.cz

*Correspondence may also be addressed to:

Evzen Boura

Tel.: +420220183465

Email: boura@uochb.cas.cz

SI Figures

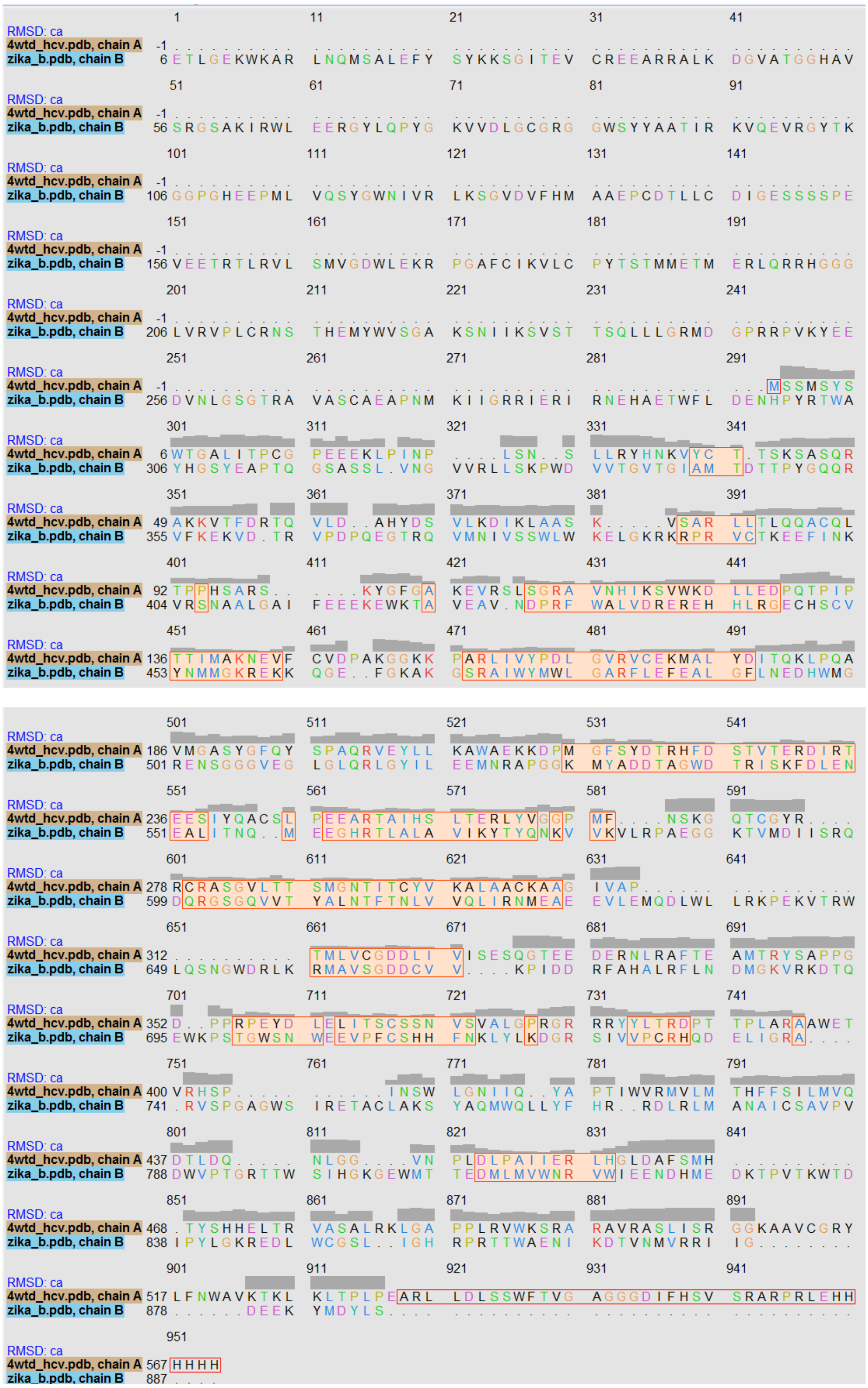

Figure S1. The report on alignment of the HCV and ZIKV RdRp structures by using the Chimera. The 174 amino acid pairs employed in the refinement are within the red boxes. The RMSD between 174 pruned atom pairs was 1.123 Å.

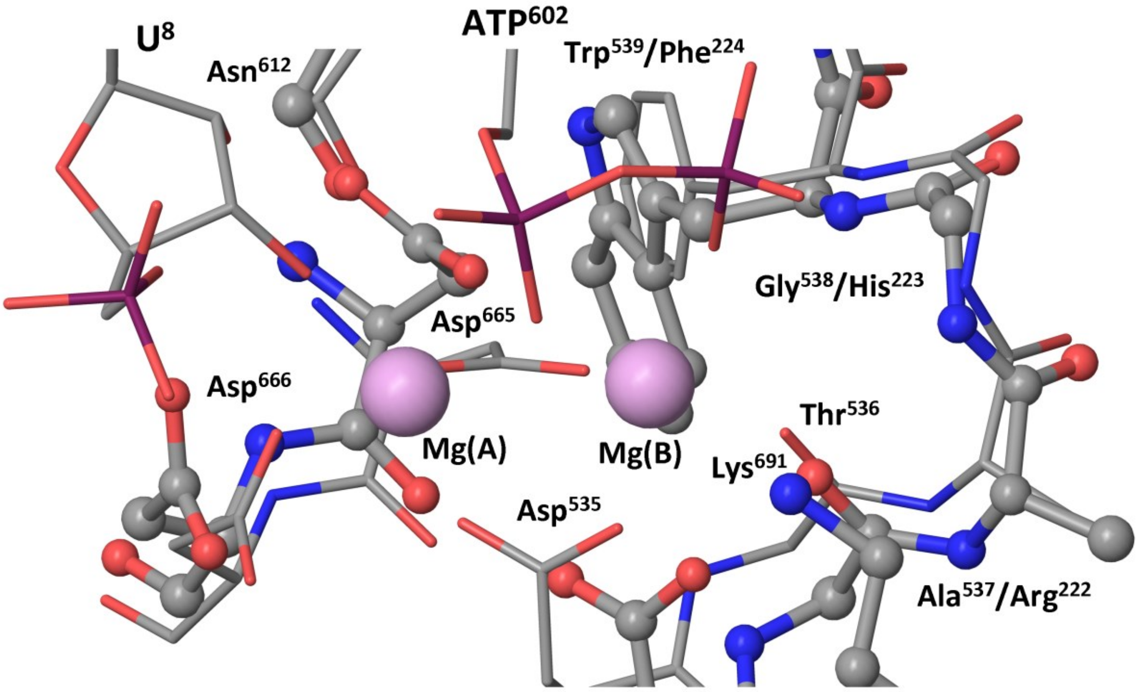

Figure S2. The alignment of catalytic core of HCV (thin-tube depiction) and ZIKV RdRp (ball & stick depiction) structures obtained using the Chimera. The Gly^{538}/His^{223}, Trp^{539}/Phe^{224}, and Ala^{537}/Arg^{222} denote the amino acid residues in ZIKV/HCV, respectively.

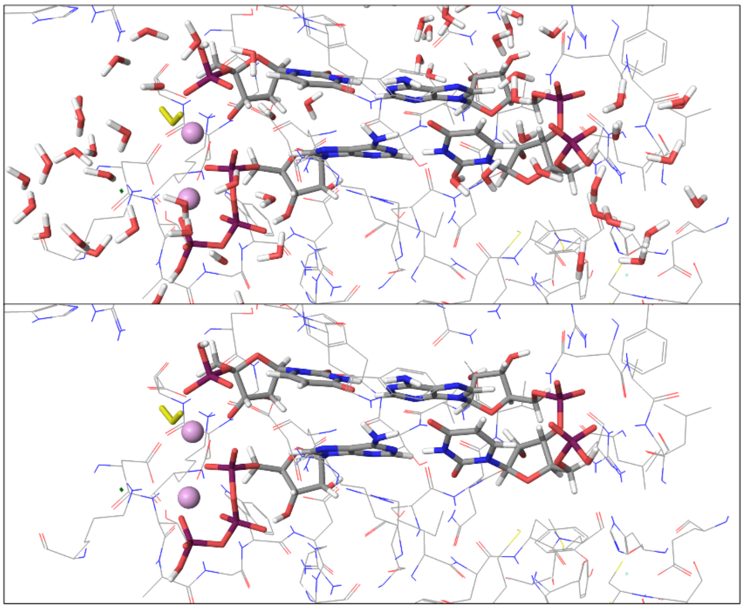

Figure S3. Hydration of the catalytic core within the solvated structural model (upper sketch) and hydration of the catalytic core within the initial structural model that included one x-ray-resolved water molecule colored in yellow (bottom sketch). The balls represent two Mg^{2+} atoms.

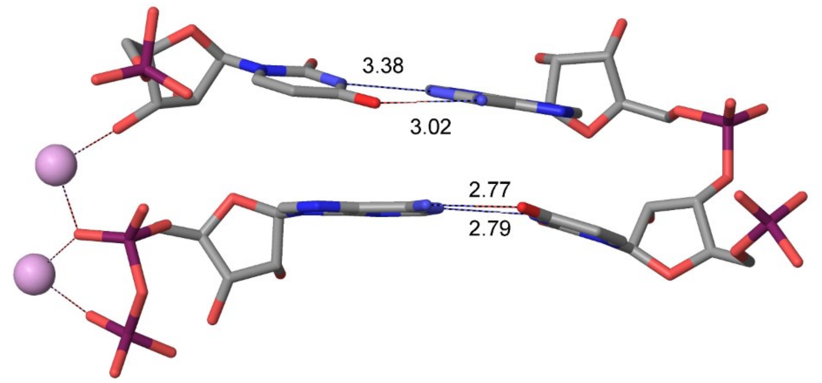

Figure S4. Sketch of the RNA molecules and Mn^{2+} cations within the catalytic core including U^8-A^3 (upper base pair) and ADP^{602}-U^2 (bottom base pair) captured in the HCV crystal (PDB ID 4WTD). The H-bonding within the base pairs and coordination of the metal cations to RNA are illustrated with dashed lines.

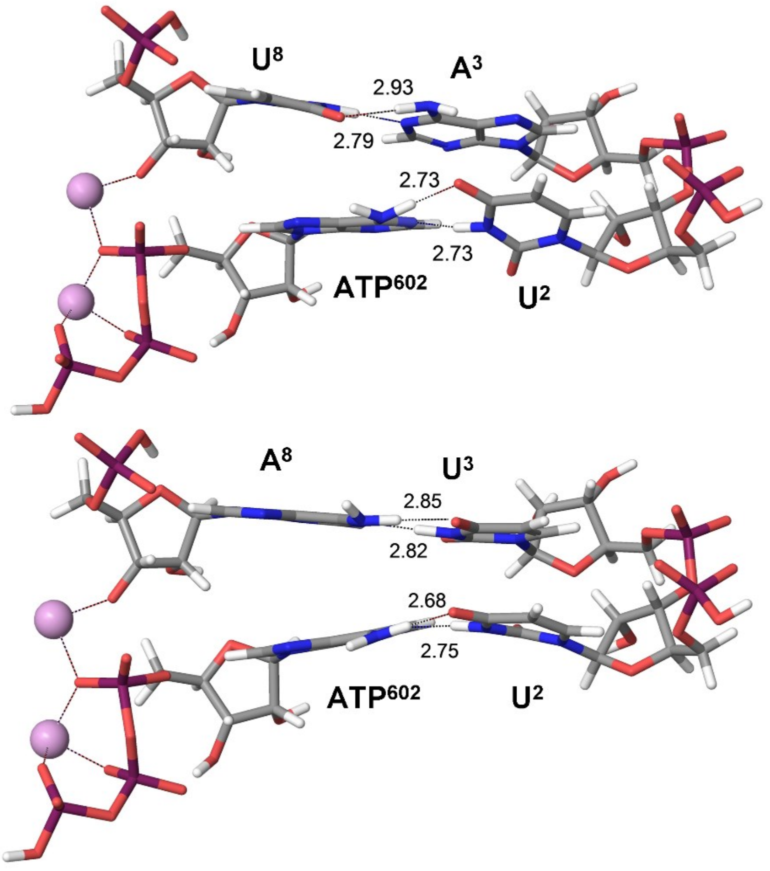

Figure S5. Sketch of the RNA molecules and Mg^{2+} cations within the catalytic core including U^8-A^3 (upper figure) and A^8-U^3 (bottom figure) with ATP^{602}-U^2 base pair, which were QM/MM-calculated for O3'-deprotonated U^8 and O3'-deprotonated A^8, respectively. The H-bonding within base pairs and coordination of metal cations to RNA are illustrated with dashed lines. The N-N and N-O distances within H-bonds are in Å.

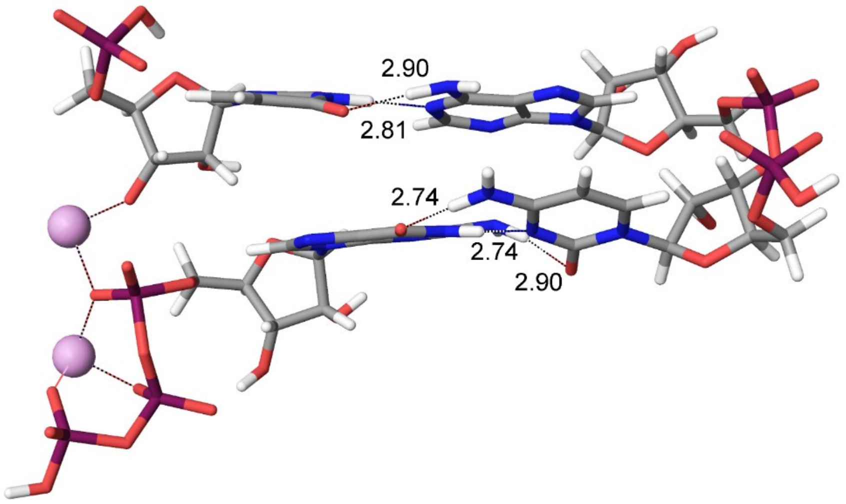

Figure S6. Sketch of the RNA molecules and Mg^{2+} cations within catalytic core including U^8-A^3 (upper base pair) and GTP^{602}-C^2 (bottom base pair) base pair QM/MM-calculated for O3'-deprotonated U^8. The H-bonding within base pairs and coordination of metal cations to RNA are illustrated with dashed lines. The N-N and N-O distances within H-bonds are in Å.

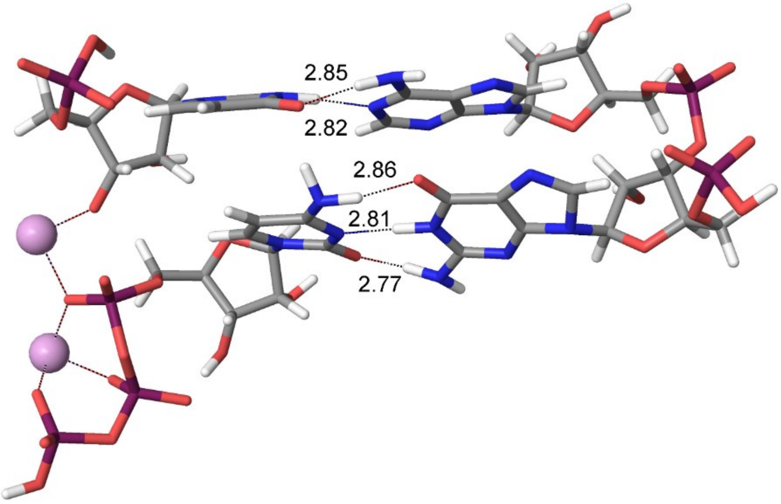

Figure S7. Sketch of the RNA molecules and Mg^{2+} cations within catalytic core including U^8-A^3 (upper base pair) and CTP^{602}-G^2 (bottom base pair) base pair QM/MM-calculated for O3'-deprotonated U^8. The H-bonding within base pairs and coordination of metal cations to RNA are illustrated with dashed lines. The N-N and N-O distances within H-bonds are in Å.

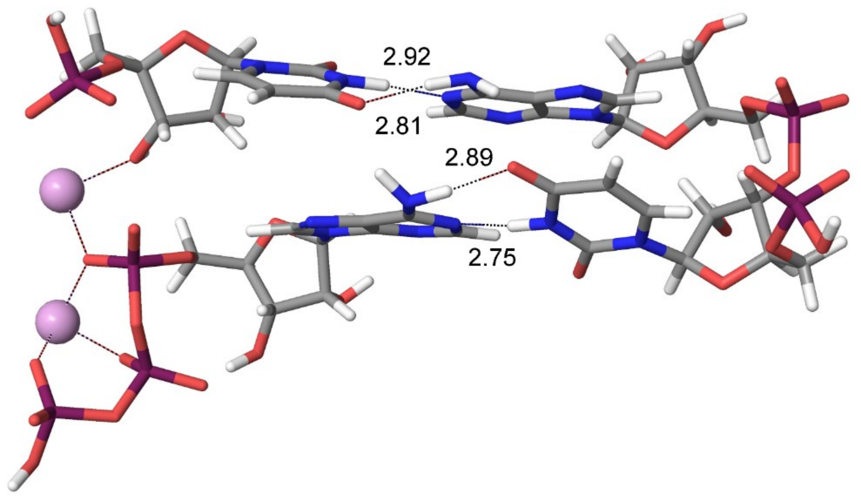

Figure S8. Sketch of the RNA molecules and Mg^{2+} cations within catalytic core including U^8-A^3 (upper base pair) and ATP^{602}-U^2 (bottom base pair) base pair QM/MM-calculated for normal U^8. The H-bonding within base pairs and coordination of metal cations to RNA are illustrated with dashed lines. The N-N and N-O distances within H-bonds are in Å.

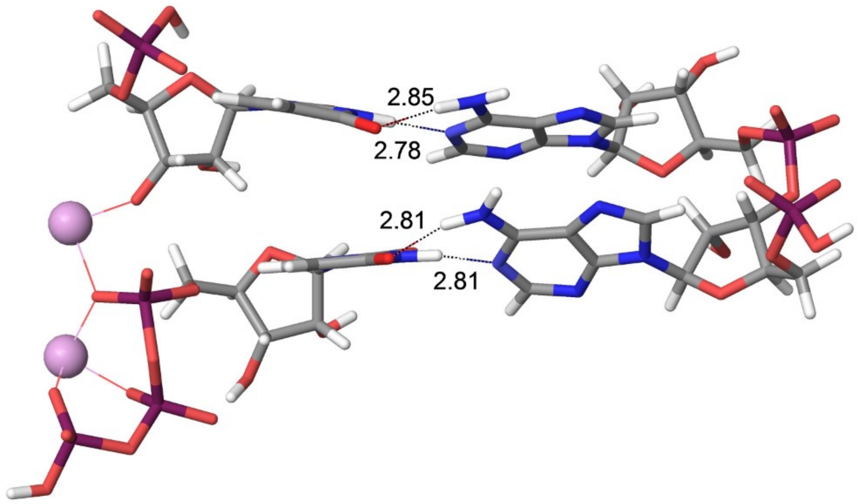

Figure S9. Sketch of the RNA molecules and Mg^{2+} cations within catalytic core including U^8-A^3 (upper base pair) and UTP^{602}-A^2 (bottom base pair) base pair QM/MM-calculated for O3'-deprotonated U^8. The H-bonding within base pairs and coordination of metal cations to RNA are illustrated with dashed lines. The N-N and N-O distances within H-bonds are in Å.

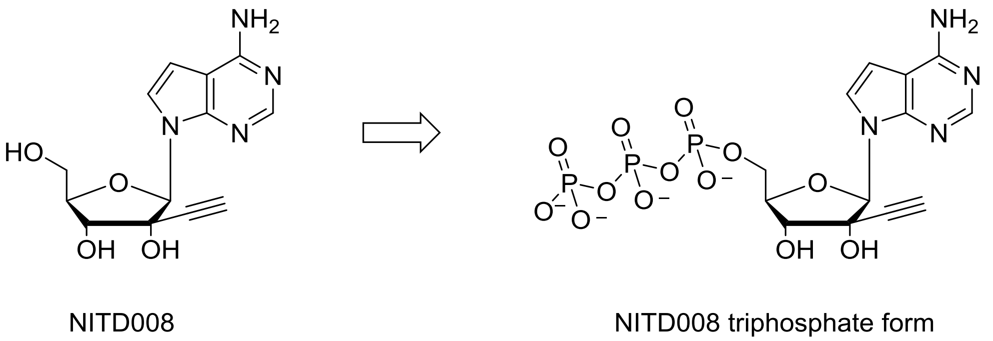

Figure S10. The structure of NITD008 and its triphosphate form.

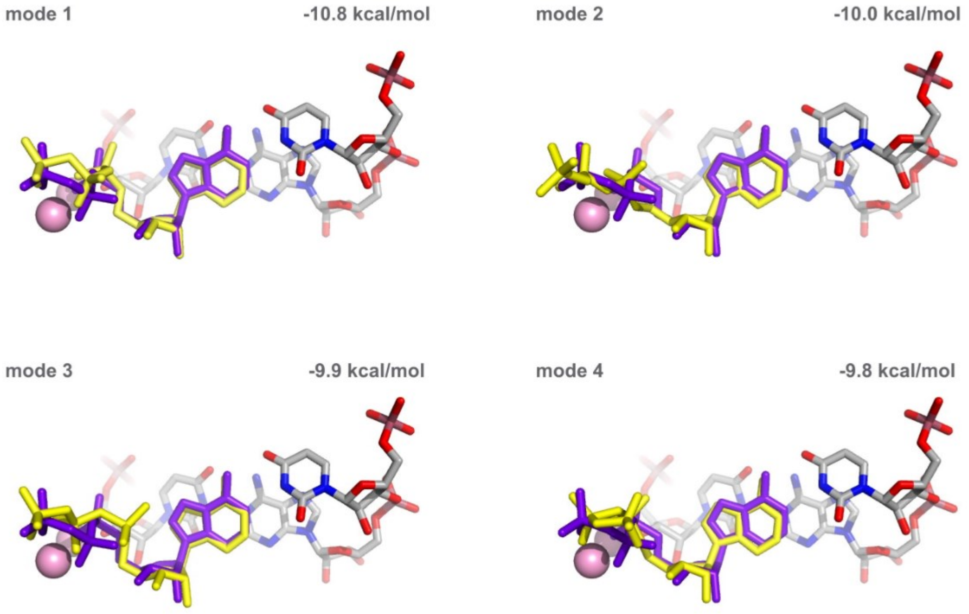

Figure S11.
Results of the docking studies – ATP docking modes with lowest energies.

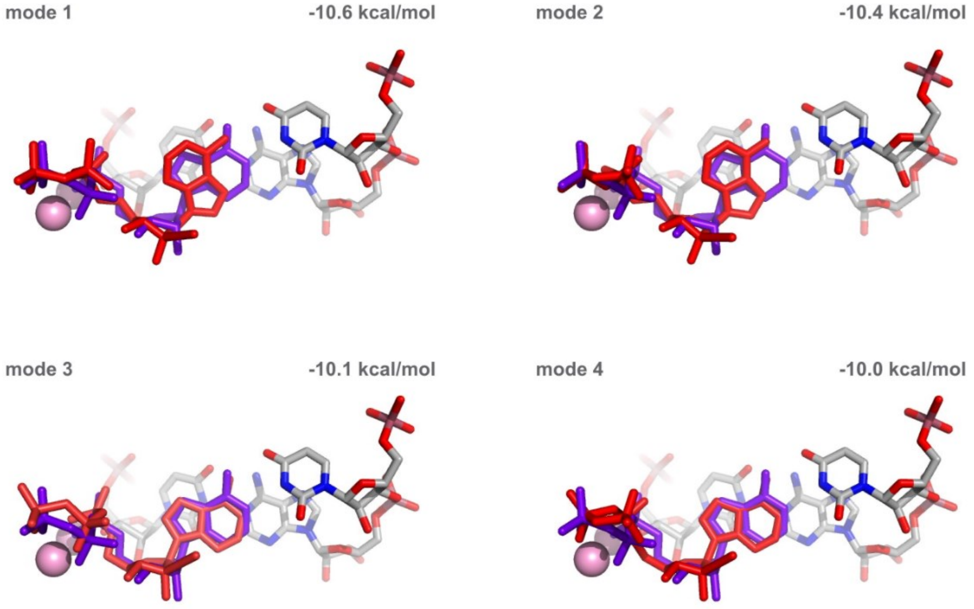

Figure S12.
Results of the docking studies –lowest energies of NIDT008 in North conformation.

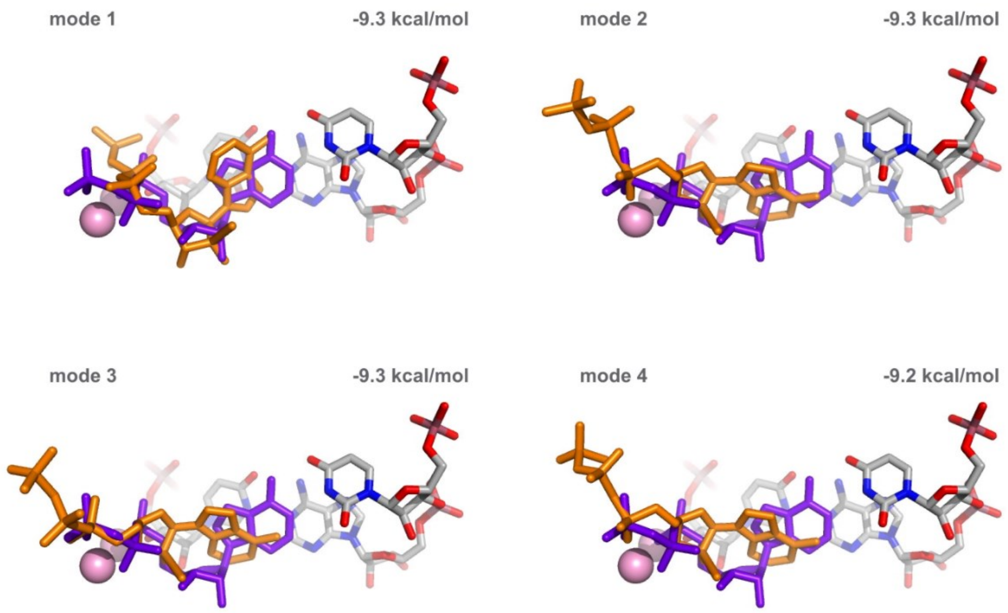

Figure S13.
Results of the docking studies –lowest energies of NIDT008 in South conformation.

SI Tables

Table S1. The comparison of torsion angles within template RNA backbone including nucleotides U^2 and A^3 with the torsion angles in canonical A-RNA. The torsions are in degrees.

	U^2						A^3			
	α	β	γ	δ	ε	ζ	α	β	γ	δ
HCV(4WTD)	267	232	64	76	194	299	286	176	54	84
A-RNARef.[22]	295	173	54	80	210	287	295	173	54	80

Table S2. The interatomic distances between metal cation (Mg^{2+} in QM/MM calculations and Mn^{2+} in 4WTD) and the linked atoms in metal-ligands in Å. The QM/MM calculations for all four alternatives of RNA base pairs involving accessing nucleotide-tri-phosphate and paired nucleotide number 2 within template RNA employed the structural model including O3'-deprotonated U^8. The terminus of nascent RNA U^8 was paired with the A^3 of template RNA in all calculations and also in the 4WTD. For numbering of residues within catalytic core see the Figure 1.

Interatomic Distance [Å]	QM/MM					X-ray (4WTD)
	ATP^{602}-U^2	UTP^{602}-A^2	GTP^{602}-C^2	CTP^{602}-G^2	ATP^{602}-U^2	ADP^{602}-U^2
	U^8[O3']-A(3)	U^8[O3']-A^3	U^8[O3']-A^3	U^8[O3']-A^3	A^8[O3']-U^3	U^8-A^3
O3'U^8 – M(A)	1.973	1.974	1.970	1.975	1.977	2.129
Ow – M(A)	2.195	2.194	2.198	2.189	2.183	2.266
M(A) – M(B)	3.695	3.703	3.688	3.673	3.719	3.332
Oδ1(Asp^{666}) – M(A)	2.075	2.071	2.075	2.063	2.075	1.984
Oδ1(Asp^{535}) – M(A)	2.128	2.133	2.122	2.120	2.126	1.980
Oδ1($Asp^{665)}$ – M(A)	2.052	2.056	2.049	2.043	2.055	2.207
O1α – M(A)	2.241	2.246	2.230	2.224	2.240	2.106
O1α – M(B)	2.223	2.232	2.217	2.223	2.279	2.002
O2β – M(B)	2.087	2.075	2.080	2.083	2.068	2.056
O1γ – M(B)	2.152	2.162	2.146	2.159	2.149	-
O(Thr^{536}) – M(B)	2.133	2.125	2.141	2.133	2.128	2.123
Oδ2 (Asp^{535}) – M(B)	2.059	2.045	2.058	2.050	2.042	2.139
Oδ2 (Asp^{665}) – M(B)	2.123	2.133	2.122	2.125	2.123	2.060

Table S3. The interatomic distances between metal cation and the linked atom within metal-ligand. The catalytic core included ATP[602]-U[2] and U[8]-A[3] base pairs. The O3'H-form or O3'-form of nucleotide U[8] employed in QM/MM calculations is given in brackets. For numbering of residues see the Figure 1. Note, the calculation model included ATP[602] while the 4WTD x-ray structure included ADP[602].

Interatomic Distance [Å]	QM/MM			X-ray (4WTD)
	U[8][O3'H]		U[8][O3']	
	Mg^{2+}	Mn^{2+}	Mn^{2+}	Mn^{2+}
O3'-M(A)	2.194	2.280	1.986	2.129
O3'- Pα	3.142	3.268	3.076	3.366
Ow- M(A)	2.101	2.194	2.319	2.266
M(A)-M(B)	3.588	3.624	3.722	3.332
Oδ1(Asp[666]) - M(A)	2.069	2.119	2.153	1.984
Oδ1(Asp[535]) - M(A)	2.064	2.122	2.244	1.980
Oδ1(Asp[665]) - M(A)	2.119	2.220	2.133	2.207
O1α – M(A)	2.135	2.208	2.305	2.106
O1α – M(B)	2.204	2.249	2.266	2.002
O2β – M(B)	2.088	2.161	2.163	2.056
O1γ – M(B)	2.135	2.212	2.261	-
O(Thr[536]) – M(B)	2.169	2.223	2.200	2.123
O3α - Pα	1.678	1.677	1.688	1.611
Oδ2 (Asp[535]) – M(B)	2.060	2.115	2.092	2.139
Oδ2 (Asp[665]) – M(B)	2.083	2.143	2.205	2.060

Table S4. The propeller-twist of nucleobases within RNA base pairs in degrees.

Base pair	Base declination
QM/MM	
ATP[602]-U[2]	30
U[8][O3']-A[3]	21
ATP[602]-U[2]	21
A[8][O3']-U[3]	8
CTP[602]-G[2]	23
U[8][O3']-A[3]	24
GTP[602]-C[2]	27
U[8][O3']-A[3]	21
UTP[602]-A[2]	41
U[8][O3']-A[3]	24
ATP[602]-U[2]	29
U[8][O3'H]-A[3]	29
X-ray (4WTD) [1)]	
ATP[602]-U[2]	17
U[8]-A[3]	11

[1)] The buckle-type declination of base-planes was found in ATP[602]-U[2]. The U[8]-A[3] was propeller-twisted, but the nucleobases were planar. The QM/MM-calculated base pairs were propeller-twisted with propeller twist and out-of-plane deviations of the atoms within nucleobases.

Table S5. Comparison of QM/MM calculated geometry parameters describing interactions of RNA with protein, and O3α – Pα distance inside RNA(NTP), and O3'- Pα distance between RNA(nascent) and RNA(NTP) with distances for QM/MM-MODEL that was obtained as overlap of PDB structures ZIKA 5TFR and HCV 4WTD. The QM/MM calculations employed Mg^{2+} while the Mn^{2+} was captured in 4WTD.

Interatomic Distances<br>[Å]	ATP^{602}–U^{2}<br>U^{8}[O3']–A^{3}	ATP^{602}–U^{2}<br>U^{8}[O3'H]–A^{3}	UTP^{602}–A^{2}<br>U^{8}[O3']–A^{3}	GTP^{602}–C^{2}<br>U^{8}[O3']–A^{3}	CTP^{602}–G^{2}<br>U^{8}[O3']–A^{3}	ATP^{602}–U^{2}<br>A^{8}[O3']–U^{3}	X-ray<br>HCV + ZIKA	RN[1]
**RNA (NTP), N = A, U, G, C**								
O3α – Pα	1.696	1.678	1.697	1.693	1.704	1.689	1.611	-
**RNA(nascent)-RNA(NTP) = A, U, G, C**								
O3'- Pα	2.926	3.142	2.924	2.929	2.892	2.906	3.366	-
**Phosphate(RNA) - protein**								
O1γ^{602} – Nε Lys^{691}	2.982	3.109	2.971	2.948	2.937	2.978	4.255	1292
O2β^{602} – N Gly^{538}	3.270	3.247	3.331	3.305	3.311	3.294	3.345	1139
O2β^{602} – N Trp^{539}	3.296	3.585	3.363	3.274	3.313	3.369	2.904	1140
O1β^{602} – Nε Arg^{473}	2.902	2.946	2.884	2.899	2.871	2.828	3.436<br>Nε^{473}→NH_2^{473}	1074
OP2^{8} – Nε Lys^{462}	4.058	2.798	4.583	4.206	3.964	4.079	6.332	1063
OP2^{2} – Nδ_2 Asn^{407}	2.873	2.622	2.972	2.979	2.966	2.819	4.341	1008
OP2^{3} – NH_1 Arg^{405}	2.502	2.492	2.509	2.505	2.507	2.545	6.425	1006
OP1^{2} - N Ala^{409}	2.641	2.612	2.647	2.646	2.640	2.623	2.037	1010
OP1^{3} - NH_1 Arg^{483}	2.618	2.669	2.618	2.619	2.613	2.627	1.604	1084
O2γ^{602} – Cα Ala^{538}	3.080	3.058	3.082	3.088	3.103	3.082	5.230<br>O2γ^{538}→O2β^{538}	1138
OP3^{8} – Oγ Ser^{798}	2.809	3.339<br>OP3^{8}→O4'^{8}	2.799	2.795	2.814	2.810	4.216	1399
**Base(RNA) - protein**								
N7^{602} – Nε Lys^{458}	2.815<br>N7^{602} → O4<br>U^{8}	2.906	2.798<br>N7^{602} → O4^{602}	2.915<br>N7^{602} →O6^{602}	3.035<br>N7^{602} →N4^{602}	2.938	1.682	1059
O4^{8} - Cβ Trp^{797}	3.482	3.587	3.509	3.447	3.378	3.807<br>O4 U^{8}→N6^{8}	3.905	1398
O2^{2} – Cβ Ile^{475}	4.098	3.780	3.827<br>O2^{2}→O4^{602}<br>Cβ^{475}→Cγ2^{475}	3.622<br>O2^{2}→O6^{602}<br>Cβ^{475}→Cγ2^{475}	3.807<br>O2^{2}→N3^{2}	3.936<br>O2^{2}→O4^{2}<br>Cβ^{475}→Cδ1^{475}	4.563	1076
O2'^{602} - Nδ_2 Asn^{612}	3.054	3.136	3.120	3.082	3.130	3.114	3.517	1213
O2U^{8} - Cδ1 Tyr^{609}	4.033	3.641	3.980	3.830	3.584	4.295<br>O2^{8}→N3^{8}	3.837	1210
O2^{2} - Cα Gly^{604}	3.238	3.385	3.271<br>O2^{2}→N3^{2}	3.707<br>O2^{2}→O2'^{2}	3.351<br>O2^{2}→N3^{2}	2.722<br>O2^{2}→O2'^{2}<br>Cα^{604} →O^{604}	2.552	1205
C2^{602} – O Ser^{603}	3.059<br>C2^{602}→O2^{2}<br>O^{603}→Oγ^{603}	3.450	4.381	2.811<br>C2^{602}→N2^{602}	2.952<br>C2^{602}→N2^{2}	3.923[4]<br>C2^{602}→O2'^{602}<br>O^{603}→Cβ^{603}	2.815	1204
O2'^{8} – N Asp^{655}	3.388	3.861	3.359	3.365	3.332	3.445	2.891	1266

O3´3 - Nε Lys^{403}	3.109	3.071	3.121	3.128	3.086	3.061	4.897	1004

[1)] The residuum number (RN) in the PDB structure QM/MM-MODEL that was obtained as overlap of PDB structures ZIKA 5TFR and HCV 4WTD. The structural model was used as starting structure in QM/MM geometry optimizations.

Table S6. Results of the re-docking of ATP.

```
################################################################
# If you used AutoDock Vina in your work, please cite:        #
#                                                              #
# O. Trott, A. J. Olson,                                       #
# AutoDock Vina: improving the speed and accuracy of docking   #
# with a new scoring function, efficient optimization and      #
# multithreading, Journal of Computational Chemistry 31 (2010) #
# 455-461                                                      #
#                                                              #
# DOI 10.1002/jcc.21334                                        #
#                                                              #
# Please see http://vina.scripps.edu for more information.     #
################################################################

Output will be ATP_out.pdbqt
Reading input ... done.
Setting up the scoring function ... done.
Analyzing the binding site ... done.
Using random seed: 276622676
Performing search ... done.
Refining results ... done.

mode |   affinity | dist from best mode
     | (kcal/mol) | rmsd l.b.| rmsd u.b.
-----+------------+----------+----------
   1        -10.8      0.000      0.000
   2        -10.0      1.191      2.020
   3         -9.9      0.877      1.458
   4         -9.8      0.869      1.942
   5         -9.6      1.007      1.831
   6         -9.5      1.153      1.703
   7         -9.5      1.094      2.379
   8         -9.5      1.034      1.766
   9         -9.0      2.462      3.679
Writing output ... done.
```

Table S7. Results of the re-docking of ATP – expanded grid-box.

```
################################################################
# If you used AutoDock Vina in your work, please cite:        #
#                                                              #
# O. Trott, A. J. Olson,                                       #
# AutoDock Vina: improving the speed and accuracy of docking   #
# with a new scoring function, efficient optimization and      #
# multithreading, Journal of Computational Chemistry 31 (2010) #
# 455-461                                                      #
#                                                              #
# DOI 10.1002/jcc.21334                                        #
#                                                              #
# Please see http://vina.scripps.edu for more information.     #
################################################################

Output will be NTP4_out.pdbqt
Reading input ... done.
Setting up the scoring function ... done.
Analyzing the binding site ... done.
```

```
Using random seed: -989261008
Performing search ... done.
Refining results ... done.

mode |   affinity | dist from best mode
     | (kcal/mol) | rmsd l.b.| rmsd u.b.
-----+------------+----------+----------
   1        -10.8      0.000      0.000
   2         -9.8      1.170      2.180
   3         -9.4      0.974      1.869
   4         -9.3      0.931      2.084
   5         -8.9      4.948      8.040
   6         -8.8      2.091      3.729
   7         -8.7      2.805      4.199
   8         -8.6     10.934     12.787
   9         -8.5     10.634     12.369
Writing output ... done.
```

Table S8. Docking of ATP without added components (Mg^{2+}, RNA parts and waters).

```
################################################################
# If you used AutoDock Vina in your work, please cite:         #
#                                                              #
# O. Trott, A. J. Olson,                                       #
# AutoDock Vina: improving the speed and accuracy of docking   #
# with a new scoring function, efficient optimization and      #
# multithreading, Journal of Computational Chemistry 31 (2010) #
# 455-461                                                      #
#                                                              #
# DOI 10.1002/jcc.21334                                        #
#                                                              #
# Please see http://vina.scripps.edu for more information.     #
################################################################

Output will be NTP4_out.pdbqt
Reading input ... done.
Setting up the scoring function ... done.
Analyzing the binding site ... done.
Using random seed: 1750544544
Performing search ... done.
Refining results ... done.

mode |   affinity | dist from best mode
     | (kcal/mol) | rmsd l.b.| rmsd u.b.
-----+------------+----------+----------
   1         -8.0      0.000      0.000
   2         -7.9      5.184      7.089
   3         -7.8     11.937     13.711
   4         -7.7     11.568     14.066
   5         -7.7      5.478      6.678
   6         -7.7     14.436     16.565
   7         -7.7      5.496      7.569
   8         -7.6     13.822     16.267
   9         -7.6      4.617      6.905
Writing output ... done.
```

Table S9. Docking of ATP into the model without magnesium ions and waters in the active site.

```
################################################################
# If you used AutoDock Vina in your work, please cite:         #
#                                                              #
# O. Trott, A. J. Olson,                                       #
# AutoDock Vina: improving the speed and accuracy of docking   #
# with a new scoring function, efficient optimization and      #
# multithreading, Journal of Computational Chemistry 31 (2010)  #
# 455-461                                                      #
#                                                              #
# DOI 10.1002/jcc.21334                                        #
#                                                              #
# Please see http://vina.scripps.edu for more information.     #
################################################################

Output will be NTP4_out.pdbqt
Reading input ... done.
Setting up the scoring function ... done.
Analyzing the binding site ... done.
Using random seed: 1985929056
Performing search ... done.
Refining results ... done.

mode |   affinity | dist from best mode
     | (kcal/mol) | rmsd l.b.| rmsd u.b.
-----+------------+----------+----------
   1         -9.1      0.000      0.000
   2         -8.9      1.724      2.307
   3         -8.8      2.219      4.887
   4         -8.8      4.673      6.725
   5         -8.7      5.064      7.027
   6         -8.7      5.212      7.356
   7         -8.6      1.552      2.055
   8         -8.6      3.202      5.570
   9         -8.6      3.785      7.135
Writing output ... done.
```

Table S10. Docking of ATP without terminus of nascent RNA.

```
################################################################
# If you used AutoDock Vina in your work, please cite:         #
#                                                              #
# O. Trott, A. J. Olson,                                       #
# AutoDock Vina: improving the speed and accuracy of docking   #
# with a new scoring function, efficient optimization and      #
# multithreading, Journal of Computational Chemistry 31 (2010)  #
# 455-461                                                      #
#                                                              #
# DOI 10.1002/jcc.21334                                        #
#                                                              #
# Please see http://vina.scripps.edu for more information.     #
################################################################

Output will be NTP4_out.pdbqt
Reading input ... done.
Setting up the scoring function ... done.
Analyzing the binding site ... done.
```

```
Using random seed: -438235420
Performing search ... done.
Refining results ... done.

mode |   affinity | dist from best mode
     | (kcal/mol) | rmsd l.b.| rmsd u.b.
-----+-----------+---------+---------
   1         -9.4      0.000      0.000
   2         -9.2      3.027      4.474
   3         -9.2      2.415      3.637
   4         -9.1      1.920      2.285
   5         -8.9      4.261      7.281
   6         -8.9      2.180      3.717
   7         -8.9      2.389      3.396
   8         -8.9      4.145      5.678
   9         -8.8      2.536      4.943
Writing output ... done.
```

Table S11. Docking of ATP without both RNA components.

```
################################################################
# If you used AutoDock Vina in your work, please cite:          #
#                                                               #
# O. Trott, A. J. Olson,                                        #
# AutoDock Vina: improving the speed and accuracy of docking    #
# with a new scoring function, efficient optimization and       #
# multithreading, Journal of Computational Chemistry 31 (2010)  #
# 455-461                                                       #
#                                                               #
# DOI 10.1002/jcc.21334                                         #
#                                                               #
# Please see http://vina.scripps.edu for more information.      #
################################################################

Output will be NTP4_out.pdbqt
Reading input ... done.
Setting up the scoring function ... done.
Analyzing the binding site ... done.
Using random seed: 1750241816
Performing search ... done.
Refining results ... done.

mode |   affinity | dist from best mode
     | (kcal/mol) | rmsd l.b.| rmsd u.b.
-----+-----------+---------+---------
   1         -9.2      0.000      0.000
   2         -8.7      2.326      5.364
   3         -8.6      1.952      2.479
   4         -8.5      2.214      3.468
   5         -8.5      2.958      4.360
   6         -8.5      1.994      5.215
   7         -8.4      2.451      4.655
   8         -8.4      1.282      1.873
   9         -8.4      2.440      4.305
Writing output ... done.
```

Table S12. Docking of ATP with Arg[473] mutated to glycine.

```
################################################################
# If you used AutoDock Vina in your work, please cite:         #
#                                                              #
# O. Trott, A. J. Olson,                                       #
# AutoDock Vina: improving the speed and accuracy of docking   #
# with a new scoring function, efficient optimization and      #
# multithreading, Journal of Computational Chemistry 31 (2010) #
# 455-461                                                      #
#                                                              #
# DOI 10.1002/jcc.21334                                        #
#                                                              #
# Please see http://vina.scripps.edu for more information.     #
################################################################

Output will be NTP4_out.pdbqt
Reading input ... done.
Setting up the scoring function ... done.
Analyzing the binding site ... done.
Using random seed: 1729621252
Performing search ... done.
Refining results ... done.

mode |   affinity | dist from best mode
     | (kcal/mol) | rmsd l.b.| rmsd u.b.
-----+------------+----------+----------
   1         -9.7      0.000      0.000
   2         -9.7      1.194      2.228
   3         -9.5      1.010      1.991
   4         -9.5      1.782      2.876
   5         -9.3      1.527      3.545
   6         -9.1      3.803      5.958
   7         -9.1      2.399      4.451
   8         -9.1      1.640      2.724
   9         -9.1      3.598      5.933
Writing output ... done.
```

Table S13. Docking of ATP with Lys[458] mutated to glycine.

```
################################################################
# If you used AutoDock Vina in your work, please cite:         #
#                                                              #
# O. Trott, A. J. Olson,                                       #
# AutoDock Vina: improving the speed and accuracy of docking   #
# with a new scoring function, efficient optimization and      #
# multithreading, Journal of Computational Chemistry 31 (2010) #
# 455-461                                                      #
#                                                              #
# DOI 10.1002/jcc.21334                                        #
#                                                              #
# Please see http://vina.scripps.edu for more information.     #
################################################################

Output will be NTP4_out.pdbqt
Reading input ... done.
Setting up the scoring function ... done.
```

```
Analyzing the binding site ... done.
Using random seed: -365061996
Performing search ... done.
Refining results ... done.

mode |   affinity | dist from best mode
     | (kcal/mol) | rmsd l.b.| rmsd u.b.
-----+------------+----------+----------
   1        -10.7      0.000      0.000
   2        -10.1      1.051      1.978
   3         -9.6      2.779      4.418
   4         -9.4      1.535      2.999
   5         -9.2      2.916      4.599
   6         -9.1      4.710      6.339
   7         -9.1      4.993      6.837
   8         -9.0      4.592      6.202
   9         -9.0      2.408      3.645
Writing output ... done.
```

Table S14. NITD008 (in North conformation) - the results of the docking

```
#################################################################
# If you used AutoDock Vina in your work, please cite:          #
#                                                               #
# O. Trott, A. J. Olson,                                        #
# AutoDock Vina: improving the speed and accuracy of docking    #
# with a new scoring function, efficient optimization and       #
# multithreading, Journal of Computational Chemistry 31 (2010)  #
# 455-461                                                       #
#                                                               #
# DOI 10.1002/jcc.21334                                         #
#                                                               #
# Please see http://vina.scripps.edu for more information.      #
#################################################################

Output will be NITD008N_out.pdbqt
Reading input ... done.
Setting up the scoring function ... done.
Analyzing the binding site ... done.
Using random seed: 978716376
Performing search ... done.
Refining results ... done.

mode |   affinity | dist from best mode
     | (kcal/mol) | rmsd l.b.| rmsd u.b.
-----+------------+----------+----------
   1        -10.6      0.000      0.000
   2        -10.4      0.673      1.839
   3        -10.1      1.158      1.815
   4        -10.0      1.116      2.447
   5         -9.8      0.957      1.789
   6         -9.6      1.503      2.755
   7         -9.4      2.193      3.676
   8         -9.4      1.390      2.716
   9         -9.3      1.662      2.399
Writing output ... done.
```

Table S15. NITD008 (in South conformation) - the results of the docking

```
################################################################
# If you used AutoDock Vina in your work, please cite:          #
#                                                               #
# O. Trott, A. J. Olson,                                        #
# AutoDock Vina: improving the speed and accuracy of docking    #
# with a new scoring function, efficient optimization and       #
# multithreading, Journal of Computational Chemistry 31 (2010)  #
# 455-461                                                       #
#                                                               #
# DOI 10.1002/jcc.21334                                         #
#                                                               #
# Please see http://vina.scripps.edu for more information.      #
################################################################

Output will be NITD008S_out.pdbqt
Reading input ... done.
Setting up the scoring function ... done.
Analyzing the binding site ... done.
Using random seed: -1206744528
Performing search ... done.
Refining results ... done.

mode |   affinity | dist from best mode
     | (kcal/mol) | rmsd l.b.| rmsd u.b.
-----+------------+----------+----------
   1         -9.3      0.000      0.000
   2         -9.3      3.193      4.842
   3         -9.3      3.056      5.443
   4         -9.2      2.902      4.288
   5         -9.2      2.595      4.574
   6         -9.1      2.254      4.250
   7         -9.0      2.470      4.209
   8         -8.9      2.389      3.437
   9         -8.9      2.035      3.480
Writing output ... done.
```
